# Supplementary material for: Is increasing urbanicity associated with changes in breastfeeding duration in rural India? An analysis of cross-sectional household data from the Andhra Pradesh children and parents study
Source: BMJ Open. 2017 Sep 21;7(9):e016331. doi: 10.1136/bmjopen-2017-016331 (PMC5623574; doi:10.1136/bmjopen-2017-016331)
Supplement: Supplementary material 1 [file bmjopen-2017-016331supp001.pdf]

Supplementary table 1. Characteristics of children under 6 in the APCAPS household survey, by missing/non-missing information on breastfeeding history (n=9,312)

		Children <6 years (n=9,312) Information on current/past breastfeeding available				
		Yes (non-missing) (n=7,848)		No (missing) (n=1,464)		
		**n**	**(%)**	**n**	**(%)**	*p value*
INDIVIDUAL LEVEL						
**Infant sex**	Male	4011	(51.1)	773	(52.8)	*0.23*
	Female	3837	(48.9)	691	(47.2)	
**Age of infant**	0-1	2445	(31.2)	445	(30.4)	
	2-3	2776	(35.4)	501	(34.2)	*0.36*
	4-5	2627	(33.5)	518	(35.4)	
HOUSEHOLD LEVEL						
**Number of under 6s in household**	1	3192	(40.7)	616	(42.1)	
	2	3846	(49.0)	702	(48.0)	*0.71*
	≥3	810	(10.3)	146	(10.0)	
**Standard of living (SLI) index**	Poorest	1172	(14.9)	145	(15.5)	
	Poorer	1447	(18.4)	176	(18.8)	
	Middle	1654	(21.1)	211	(22.5)	*0.37*
	Richer	1777	(22.6)	185	(19.8)	
	Richest	1796	(22.9)	219	(23.4)	
	Missing	*2*		*528*		
VILLAGE LEVEL						
**Night-time light intensity (NTLI)**	Low	1484	(18.9)	271	(18.5)	
	Medium	2086	(26.6)	353	(24.1)	*0.09*
	High	4278	(54.5)	840	(57.4)	

Supplementary figure 1. Questions on breastfeeding practices from APCAPS cross-sectional household survey

**12.**	**Health Profile of the child (≤5 years)**							
(12.1) **Name of the Child**	(12.2) **Colostrum Intake** (1. Yes; 2. No)	(12.3) **Total Duration of Breast Feeding** (in months)	(12.4) **Age of child at onset of weaning** (in months)	(12.5) **Immunization** (1. Completed; 2. Partial; 3. None)	(12.6) **Normal Healthy Child** (1. Yes; 2. No)	**Child Supplemented during** (1. Yes; 2. No; 3. N.A.)		
						(12.7.1) **Infancy**	(12.7.2) **Childhood**	(12.7.3) **Current**
