# Supplementary material for: Is increasing urbanicity associated with changes in breastfeeding duration in rural India? An analysis of cross-sectional household data from the Andhra Pradesh children and parents study
Source: BMJ Open. 2017 Sep 21;7(9):e016331. doi: 10.1136/bmjopen-2017-016331 (PMC5623574; doi:10.1136/bmjopen-2017-016331)
Supplement: Author's manuscript [file bmjopen-2017-016331.draft_revisions.pdf]

Is increasing urbanicity associated with changes in breastfeeding duration in rural India? An analysis from the Andhra Pradesh Children and Parents Study

Journal:	*BMJ Open*
Manuscript ID	bmjopen-2017-016331
Article Type:	Research
Date Submitted by the Author:	08-Feb-2017

Complete List of Authors:	Oakley, Laura; London School of Hygiene and Tropical Medicine, Non-communicable Disease Epidemiology<br>Baker , Chris; London School of Hygiene and Tropical Medicine, Non-communicable Disease Epidemiology<br>Addanki, Srivalli; Indian Institute of Public Health<br>Gupta, Vipin; University of Delhi, Department of Anthropology<br>Walia, Gagandeep; Public Health Foundation of India, Centre for Control of Chronic Conditions<br>Aggarwal, Aastha; Public Health Foundation of India, Centre for Control of Chronic Conditions<br>Bhogadi, Santhi; Indian Institute of Public Health<br>Kulkarni, Bharati; National Institute of Nutrition<br>Wilson, Robin; University of Southampton, Geography & Environment<br>Prabhakaran, Dorairaj; Public Health Foundation of India, Centre for Control of Chronic Conditions<br>Ben-Shlomo, Yoav; University of Bristol, School of Social and Community Medicine<br>Davey Smith, George; University of Bristol, School of Social and Community Medicine<br>Radha Krishna, KV; National Institute of Nutrition<br>Kinra, Sanjay; London School of Hygiene and Tropical Medicine, Non-communicable Disease Epidemiology

**Primary Subject Heading**:	Epidemiology
Secondary Subject Heading:	Global health
Keywords:	SOCIAL MEDICINE, NUTRITION & DIETETICS, PUBLIC HEALTH

SCHOLARONE™
Manuscripts

Is increasing urbanicity associated with changes in breastfeeding duration in rural India? An analysis from the Andhra Pradesh Children and Parents Study

Running title: Urbanicity and breastfeeding in rural India

Oakley L[1]*, Baker C[1], Addanki S[2], Gupta V[3], Walia GK[4], Aggarwal A[4], Bhogadi S[2], Kulkarni B[5], Wilson RT[6], Prabhakaran D[4], Ben-Shlomo Y[7], Smith GD[7], Radha Krishna KV[5], Kinra S[1]

[1]Department of Non-communicable Disease Epidemiology, London School of Hygiene, London, UK.

[2]Indian Institute of Public Health, Hyderabad, India.

[3]Department of Anthropology, University of Delhi, India.

[4]Centre for Control of Chronic Conditions, Public Health Foundation of India, New Delhi, India

[5]National Institute of Nutrition, Hyderabad, India.

[6]Geography & Environment, University of Southampton, Southampton, UK.

[7]School of Social and Community Medicine, University of Bristol, Bristol, UK.

*Corresponding author
Dr Laura Oakley, Assistant Professor, Department of Non-communicable Disease Epidemiology, London School of Hygiene and Tropical Medicine, Keppel St, London WC1E 7HT (laura.oakley@lshtm.ac.uk)

Main text word count: 3729 words

For peer review only - http://bmjopen.bmj.com/site/about/guidelines.xhtml

ABSTRACT

Objective: To investigate whether village-level urbanicity and lower-level socio-economic factors are associated with breastfeeding practices in transitioning rural communities in India.

Setting: 29 villages in Rangareddy district, southern India between 2011-2014.

Participants: 7,848 children under 6 years identified via a cross-sectional household survey conducted as part of the Andhra Pradesh Children and Parents Study.

Outcome measures: Two key indicators of optimal breastfeeding: termination of exclusive breastfeeding before six months and discontinuation of breastfeeding by 24 months. Village urbanicity was classified as low, medium or high according to satellite assessed night-light intensity.

Results: Breastfeeding initiation was almost universal, and approximately two in three children were exclusively breastfed to six months and a similar proportion breastfed to 24 months. Using multilevel logistic regression, increasing urbanicity was associated with breastfeeding discontinuation before 24 months (medium urbanicity OR 1.45, 95% CI 0.71-2.96; high urbanicity OR 2.96, 95% CI 1.45-6.05) but not with early (<6 months) termination of exclusive breastfeeding. Increased maternal education was independently associated with both measures of suboptimal breastfeeding, and higher household socio-economic position was associated with early termination of exclusive breastfeeding.

Conclusion: In this transitional Indian rural community, early stage urbanicity was associated with a shorter duration of breastfeeding. Closer surveillance of changes in breastfeeding practices alongside appropriate intervention strategies are recommended for emerging economies.

For peer review only

For peer review only - http://bmjopen.bmj.com/site/about/guidelines.xhtml

ARTICLE SUMMARY

- Previous studies have investigated the association between urbanisation and breastfeeding using the urban-rural dichotomy.
- We used data from a large rural cohort in southern India that is currently undergoing rapid and uneven urbanisation due to its proximity to a major urban centre.
- The use of night-time light intensity data as an indicator of urbanicity allowed us to examine subtler changes in breastfeeding practices along the urban-rural continuum.
- Sixteen percent of children were excluded from the analysis due to missing information on breastfeeding practices.
- We relied on maternal retrospective recall of breastfeeding events for our outcome measurement.

For peer review only

For peer review only - http://bmjopen.bmj.com/site/about/guidelines.xhtml

INTRODUCTION

The promotion of breastfeeding is one of the three interventions identified as having the largest potential impact on global child death[1]. Optimal breastfeeding is defined by the World Health Organisation (WHO) as early breastfeeding initiation, exclusive breastfeeding to six months, and continued breastfeeding to two years or beyond alongside appropriate complementary feeding. Many low and middle income countries (LMICs) have a strong tradition of near universal and prolonged breastfeeding[2, 3], though exclusive breastfeeding to six months (hereafter referred to simply as 'exclusive breastfeeding') is less common. A small increase in the global proportion of children exclusively breastfed between 1995 and 2010 has been reported, but the overall proportion (40%) still falls strikingly short of universal coverage and obscures differences in country-specific trends[4].

Many LMICs are currently experiencing a rapid increase in the proportion of people living in built-up areas, and the social, cultural and economic changes associated with this process of urbanisation have the potential to impact on traditional breastfeeding practices. Direct threats to optimal breastfeeding include early introduction of other liquids, and inappropriate supplementation with solid or semi-solid foods. These behaviours may be influenced by changing social norms, for example increasing numbers of mothers working outside the home. Of all positive health behaviours, breastfeeding is one of the few more prevalent in LMICs compared to HICs[3]. Within LMICs this trend is mirrored by a higher prevalence of suboptimal breastfeeding in urban areas compared to rural areas[5]: a trend also observed in India[6-8] alongside variation by various socio-economic indicators [6-10]. Although the high level urban-rural comparison is of interest, there may also be subtler changes in breastfeeding practices along the urban-rural continuum given the peri-urban effects on villages close to urban centres. These changes can potentially be investigated by using a measure of 'urbanicity' which aims to assess the extent of urbanisation in a given area. A number of different indicators of urbanicity have evolved, including the use of remote light sensing[11, 12], and

For peer review only - http://bmjopen.bmj.com/site/about/guidelines.xhtml

multi-component scales[13, 14]. The early identification of changes in breastfeeding practices accompanying the urbanicity transition - and an understanding of the underlying mechanisms - are necessary for informing appropriate interventions to protect traditionally positive breastfeeding practices in transitioning communities.

The Andhra Pradesh Children and Parents Study (APCAPS) is a rural socio-demographic cohort in southern India that is currently undergoing rapid and uneven urbanisation due to its proximity to a major urban centre (Hyderabad), providing a unique opportunity to examine the association between early stage urbanicity and breastfeeding practices.

METHODS

Study design

APCAPS is an intergenerational cohort originally established to study the long-term effects of early-life undernutrition on risk of cardiovascular disease and subsequently expanded to include trans-generational influences of other environmental and genetic factors on chronic diseases in transitioning rural India.

The original cohort is based on the participants in the Hyderabad Nutrition Trial (HNT) conducted in 1987–90 in 29 villages approximately 50-100km from Hyderabad in Telangana state (formally Andhra Pradesh), southern India[15]. The dataset used in this analysis is based on a cross-sectional household survey conducted between 2011 and 2014 in the study villages. All households (household defined as a group of people living in the same residence and sharing a common kitchen) in the study villages were visited by fieldworkers and socio-demographic information was collected on each household. In addition, a basic health profile was collected for each child under 6 years of age, comprising information on infant feeding (colostrum intake, total duration of breastfeeding, age of onset of weaning), immunization and anthropometric measurements. Fieldworkers made repeated

For peer review only - http://bmjopen.bmj.com/site/about/guidelines.xhtml

visits to households to maximise response and to clarify inconsistencies in collected data. Data were collected from 23,314 households in total, of which 5,968 (25.6%) included at least one child under 6 years.

The study received approval from the ethics committees of the National Institute of Nutrition (NIN) (Hyderabad, India) and London School of Hygiene and Tropical Medicine (London, UK). Approval was also sought from the Indian Council for Medical Research and the village heads and their committees in each of the study villages. Written informed consent (or witnessed thumbprint if illiterate) was obtained from the participants prior to their inclusion in the study.

Breastfeeding outcomes and explanatory variables

Two breastfeeding outcomes were used in this analysis: termination of exclusive breastfeeding (EBF) before six months, and discontinuation of breastfeeding before 24 months. These outcomes reflect failure to achieve two of the specific WHO recommendations for optimum feeding practices (exclusive breastfeeding to six months and continued breastfeeding to two years)[16]. The age of the child at termination of EBF was derived from information provided by mothers on the child's age at 'weaning' (defined by fieldworkers as the age at which the child was given anything other than mother's milk, i.e. age at initiation of complementary feeding).

Our primary explanatory factor was urbanicity, measured using remotely-sensed village-level night-time light intensity (NTLI) scores, as these are objective, unbiased and easily available over wide areas. Although this analysis represents the first application of NTLI data to the APCAPS population, NTLI data is increasingly being used as an area-based indicator of socio-economic development[11, 12]. The light which is included in the NTLI score include any outside lights, ranging from fires and gas flares to lights related to human settlements. Low level lights such as from streets and car headlights can be observed if there is a sufficient number of sources, but indoor lights

For peer review only - http://bmjopen.bmj.com/site/about/guidelines.xhtml

cannot be observed. NTLI scores were calculated for 2012 using the National Oceanic and Atmospheric Administration (NOAA) Stable Lights product which provides yearly average night-time light intensity measures processed and filtered to remove events such as fires and lightening contamination by cloud or moon reflections and background noise, at a 1km resolution. Scores for each village were calculated by summing the raw NTLI values over each village polygon (digitised using Bing Maps combined with GPS-based surveying by the field teams). The 1km-resolution NTLI data was upscaled to 100m resolution to allow more accurate estimation of the NTLI values covered by each polygon, as many villages are small and partially cover multiple 1km grid cells.

NTLI scores for each village were validated against alternative urbanicity measurements (field worker ranking and a multi-component urbanicity score based on household-level material assets and village-level availability of infrastructure and services) showing positive correlations (0.65 and 0.53 respectively) . Study villages were ranked by their NTLI score and divided into tertiles to represent 'low' (10 villages), 'medium' (10 villages) and 'high' (9 villages) levels of urbanicity. The NTLI tertile scores matched the field worker ranking in 50% of the villages, and cases of disagreement between NTLI and field worker ranking, the latter was more conservative and ranked villages as medium urbanicity rather than high urbanicity. Only one village had a significant divergence between NTLI and fieldworker ranking.

In addition, we investigated mother-level socio-economic factors which may be correlated with urbanicity: maternal education (no formal education, primary education, or secondary education and higher), maternal employment (paid work vs. no paid work), and a household level standard of living index (SLI). Asset-based SLIs have been established as a valid proxy measure of household wealth[17]. We generated a SLI score for each household, calculated by using information on household assets including house and land ownership, characteristics of the home (electricity, water pump, separate kitchen, separate toilet) and ownership of various assets (tractor, radio, AC, washing machine, bore hole, telephone, TV, fridge, bicycle, two wheeler, four wheeler, bank account, animal

For peer review only - http://bmjopen.bmj.com/site/about/guidelines.xhtml

cart, sofa, cot/bed, mattress, table). Principal component analysis (PCA) was used to determine the weights for each component in the index[18], and households were divided into quintiles according to their weighted score. We also report data on a number of other factors likely to be associated with breastfeeding practices: sex of child, birth order, maternal age (grouped), and household composition (joint/extended or nuclear).

Statistical analysis

We included in the analysis all children under 6 years who were breastfed at least once and for whom information was available on feeding history. The analysis investigating termination of EBF before six months was restricted to children six months or older at the time of survey, and correspondingly only those children aged 24 months or older were included in the analysis of discontinuation of breastfeeding before 24 months. A small proportion of children (3%) had missing information on one or more variables of interest and were excluded.

We hypothesised that urbanicity would be associated with less favourable breastfeeding practices. This could operate through at least two different indirect pathways (see figure 1): through increasing individual-level employment, education or assets so that households are less likely to maintain breastfeeding, or due to more urbanised villages have a different 'collective' attitude to breastfeeding. We investigated these hypotheses by using multilevel logistic regression modelling with children (level 1) nested within mothers (level 2, max n=5,477) nested within villages (level 3, n=29). This approach allowed us to model the variation in breastfeeding outcomes at each level (random effects), and to estimate the effect of specific mother and village-level factors on breastfeeding practices (fixed effects). We initially fitted a null model (model 1) for each of the two outcomes with random intercepts only in order to estimate the baseline between-mother and between-village variance. We then fitted a series of models for each breastfeeding outcome, adding

For peer review only - http://bmjopen.bmj.com/site/about/guidelines.xhtml

covariates as fixed effects to the included random effects, where fixed effects were interpreted as the average effect on the specified breastfeeding outcome across all mothers and villages. These models included individual demographic factors and mother-level socioeconomic indicators (model 2), individual demographic factors and village-level urbanicity (model 3), and all variables (model 4). Due to the correlation between socio-economic indicators and urbanicity, we considered estimates from model 3 our main results. Proportional change in variance (PCV) was calculated as a measure of change in mother-level (level 2) and village-level (level 3) variance between the null model and subsequent models, and (for village-level variance only) the measure of change between a model with (model 4) and without (model 2) the village-level urbanicity variable included.

Estimates of the association between mother-level socio-economic variables and breastfeeding outcomes were derived from model 2 (adjusted for individual-level demographic variables, but not urbanicity).

We hypothesised *a priori* that the association between village-level urbanicity and breastfeeding may vary by household SLI and maternal education. We investigated these cross-level interactions in further models (for SLI, comparing the richest two quintiles to the three poorest quintiles; for education, comparing secondary education versus no or primary education).

All statistical analyses were conducted using Stata 14 (StataCorp, College Station, Texas, USA).

RESULTS

Characteristics of the sample

Information on breastfeeding was available on a total of 7,848 children (5,390 households), 99% (n=7,839) of whom were breastfed at least once (figure 2).

The characteristics of ever breastfed children by urbanicity of village are presented in table 1. There was little variation in infant sex, birth order or maternal age by urbanicity of village. Children

For peer review only - http://bmjopen.bmj.com/site/about/guidelines.xhtml

residing in villages classified as more urbanised had mothers that were more likely to have been educated to secondary level, less likely to have mothers in paid employment, and a higher standard of living according. Joint/extended families were slightly less prevalent in high urbanicity villages.

Termination of EBF by six months

One third of children (33.5%, n=2,420) were EBF for a period of less than six months (table 1).

Fixed effects

There was no statistically significant trend regarding early termination of EBF and village level urbanicity. The prevalence of early termination of EBF was lowest in medium urbanicity villages (27.2%), higher in lower urbanicity villages (33.6%), and highest in high urbanicity villages (36.5%). In multivariable analysis there was no evidence that urbanicity was associated with termination of EBF by six months (model 3, table 2), with little change in estimates after the addition of demographic and socio-economic covariates to the model.

After adjustment for other individual- and mother-level covariates, both children of mothers with primary education and children of mothers with secondary education were more likely to be EBF for less than six months when compared to children of mothers with no formal education (primary education OR 3.37, 95% CI 2.13-5.31; secondary education OR 1.69, 95% CI 1.12-2.54; model 2, table 2). Increasing SLI quintile was associated with up to twice the odds of early termination of EBF compared to children from the poorest households (richest quintile OR 2.11, 95% CI 1.22-3.63; p value for trend = 0.003). There was some evidence that maternal employment was also associated with early termination of EBF (OR 1.43, 95% CI 1.00-2.03). The estimates for socio-economic variables did not change with the addition of urbanicity to the model.

Interaction effects

For peer review only

There was no evidence of interaction between urbanicity and either household SLI or maternal education.

Random effects

There was statistically significant unexplained variance estimates at both the mother and village level (model 1, table 1). Unexplained variability was consistently higher at the mother level compared to the village level. The addition of individual level parameters resulted in a slight decline in community-level variation (variance 1.50 and 1.44 in models 1 and 2 respectively). There was a further decline in village-level variance when the urbanicity variable was added to the model (variance 1.32 in model 4). Comparing the village-level variance between model 2 and model 4 suggests that 8.5% of the observed village-level variation can be explained by urbanicity.

Discontinuation of breastfeeding by 24 months

Nearly four in ten children (37.8%, n=2037) were breastfed for less than 24 months in total.

Fixed effects

Discontinuation of breastfeeding by 24 months was more common in high urbanicity villages (42.1%) and least common in low urbanicity villages (29.6%). After adjustment for individual-level demographic factors, high urbanicity was associated with increased odds of breastfeeding discontinuation before 24 months (OR 2.64, 95% CI 1.29, 5.42; model 3, table 3). The OR for medium urbanicity was slightly increased, though not statistically significant at p <0.05 (OR 1.45, 95% CI 0.71, 2.96; model 4) and there was evidence of a linear trend (p value 0.008). Additional adjustment for socio-economic variables resulted in a slight reduction in the odds ratios (high urbanicity OR 2.64, 95% CI 1.29, 5.42; medium urbanicity OR 1.35, 95% CI 0.66, 2.79; model 4).

When compared to children of mothers with no formal schooling, children of mothers with secondary education were at significantly higher odds of breastfeeding discontinuation after

For peer review only - http://bmjopen.bmj.com/site/about/guidelines.xhtml

adjustment for all demographic and socio-economic factors (OR 1.63, 95% CI 1.23-2.16; model 2, table 3). Maternal employment was associated with a slight reduction in the odds of breastfeeding discontinuation before 24 months (OR 0.77, 95% CI 0.60-0.99). There was no evidence that SLI quintile was independently associated with breastfeeding discontinuation by 24 months. The inclusion of urbanicity in the model did not alter the socio-economic estimates of effect.

Interaction effects

There was no evidence of interaction between urbanicity and mother-level socio-economic factors (household SLI and maternal education).

Random effects

The random effects parameters for models investigating discontinuation of breastfeeding before 24 months are presented in table 3. In the null model (model 1) the proportion of residual variance attributable to mothers (level 2, 53.7%) was much higher than the variance attributable to villages (level 3, 8.5%). The addition of urbanicity to a model including individual and mother-level factors resulted in a decrease of 8.5% in village-level variance.

DISCUSSION

Summary of main findings

In this study approximately two in three children were exclusively breastfed to six months and a similar proportion breastfed to 24 months. At the village level, high urbanicity was associated with breastfeeding discontinuation before 24 months, but there was no evidence that urbanicity was associated with early termination of EBF. At the mother level, increased maternal education was independently associated with both indicators of suboptimal breastfeeding, and high SLI associated

For peer review only

with an increased odds of EBF for less than 6 months. Maternal employment showed a variable association with breastfeeding. The residual variation in breastfeeding outcomes suggested greater heterogeneity within-villages than between-villages.

Consistency with previous studies

Our estimates of breastfeeding prevalence are largely consistent with those derived from other population-based studies in India. Early results from NFHS-4 (2015-16) report 67.3% of infants aged 0-6 months in Telangana are exclusively breastfed [19]. The overall proportion of children breastfed until at least 24 months in our study was almost identical to an analysis of all-India NFHS-2 data: (62.2% vs. 63%) [6].

Very few existing studies have investigated the association between urbanicity and breastfeeding. In one study based in the Philippines, Dahly et al. reported that length of breastfeeding was negatively correlated with increasing urbanicity (using a multicomponent measure)[13]. The persisting association between high urbanicity and increased odds of breastfeeding discontinuation<24 months - after adjustment for lower level socio-economic circumstances - reported in our study support the findings from Dahly et al.

Increasing urbanicity is associated with positive socio-economic changes such as improved education for women and increased income and household wealth. A number of other studies from India and other LMICs have demonstrated a negative association between improved socio-economic position and breastfeeding practices[6-8, 20-23]. We found similar results with regard to household SLI and increased maternal education, and early termination of exclusive breastfeeding. One explanation for this trend could be the greater affordability and/or social desirability of commercial breast milk substitutes. The association between education and early termination of exclusive breastfeeding was strongest for primary education (primary education OR 3.37, 95% CI 2.13-5.31;

For peer review only

secondary education OR 1.69, 95% CI 1.12-2.54). This suggests that while education in general is associated with a reduction in the length of exclusive breastfeeding, higher levels of education partially ameliorate this effect. Interestingly, there was some evidence that maternal employment had a protective effect on breastfeeding discontinuation by 24 months, though the opposite trend was observed with regard to early cessation of EBF. There is some evidence of a U-shaped association between education and women's employment in India, with paid employment outside the home common among women with little or no formal education, lower among women with moderate levels of education, and rising again with high levels of education[24]. Mothers in employment are likely to be a heterogeneous group, making it difficult to draw any firm conclusions about the association between paid employment and breastfeeding practices in this sample.

Strengths and limitations

The APCAPS cohort provides a unique opportunity to investigate current health behaviour and outcomes in a large cohort set against the backdrop of rapid urbanisation and economic transition in rural India. While studies based on the high level urban-rural comparison may help to predict the impact of 'total' urbanisation on breastfeeding practices, they obscure the temporal emergence of subtler changes in the urban environment which may be amenable to intervention. The use of multilevel models enabled us to explore the role of factors at different levels: individual, mother, and village.

Although the vast majority of all under 6s in the study villages were included in our analysis, 15.7% (n=1,464) were excluded due to missing information on feeding history due to the mother living elsewhere, travelling, or deceased. In a comparison of included and excluded children there was no evidence that infant sex, infant age, number of under 6s living in the household, or household SLI

For peer review only

For peer review only - http://bmjopen.bmj.com/site/about/guidelines.xhtml

differed by missing status (supplementary table 1). A slightly higher proportion of excluded children resided in high urbanicity villages (p 0.09).

We relied on maternal recall of breastfeeding events for our outcome measurement. The recall period was short (<6 years) and a number of studies including those conducted in LMICs have confirmed that such short-term recall is reliable, particularly for duration of breastfeeding[25, 26]

Our measure of urbanicity was derived from night-time light intensity data, information which is objective, regularly updated and free to use. Additionally, data on night-time light intensity is available over a number of years and could be used in future studies to investigate trends in urbanicity over time. However, it must be noted that urbanicity is an ecological indicator and as such may not accurately reflect individual environment, particularly given that many women may travel regularly outside their home village for work or family reasons.

Implications

Nearly a quarter (24%) of all global under-five deaths occur in India[27]. In light of the failure to achieve the Millennium Development Goal infant mortality rate (IMR) target reduction [28], a new target of reducing the IMR to 20 per 1,000 live births by 2020 has recently been proposed [29]. Early results from the latest National Family Health Survey (NFHS-4) data collected in Telegana state report a current IMR of 28 (20 in urban areas, 35 in rural areas) [19]. An increase in optimal breastfeeding practices will help to achieve improvements in infant survival, in addition to reducing the considerable burden of infant morbidity[3, 30]}. India faces an ever-increasing epidemic of chronic disease in common with many other LMICs. Several studies have suggested that breastfeeding has a protective effect on long-term outcomes such as obesity and diabetes in adulthood[31], though residual confounding is difficult to exclude[32], and the most recent data from

For peer review only - http://bmjopen.bmj.com/site/about/guidelines.xhtml

the PROBIT RCT do not support an association between breastfeeding and adiposity in late childhood[33].

A substantial proportion of infants in India are exclusively breastfed for less than the six months recommended by WHO[7, 9, 10], and a recent study reported that there has been little change in the prevalence of exclusive breastfeeding in India between 1992-1993 and 2005-2006[8]. The lack of country-specific holistic and coordinated policy programmes supporting breastfeeding has also been highlighted[34]. Therefore, research to further understand the determinants of suboptimal breastfeeding practices in India is timely.

Our findings suggest that in LMICs with a strong tradition of breastfeeding, negative changes in breastfeeding behaviour may be observed during early stages of the urbanicity transition. Reduced duration of breastfeeding among more educated mothers may be one of the earliest markers of this change. India is currently undergoing rapid urbanisation, with the proportion of the population living in towns and cities is set to increase from an estimated 28% in 2011 to 38% by 2026[35]. Many more individuals live in areas which though traditionally described as rural are increasingly displaying many of the characteristics of urban areas. There is good evidence that breastfeeding behaviours are amenable to change through interventions delivered at the household and community level, as well as those targeting health systems[36, 37]. Intervention programmes to protect and promote breastfeeding should be considered in transitioning communities to counteract changes in breastfeeding practices, preferably targeted at those mothers identified as most at risk of suboptimal breastfeeding practices.

For peer review only

Acknowledgements

We wish to acknowledge our dedicated field teams led by Santhi Bhogadi and the study participants who made this study possible. We also acknowledge the contribution of Naveen Chittaluri and Ekta Jain to data processing and management. We also thank Cono Ariti at the London School of Hygiene and Tropical Medicine (LSHTM) who provided statistical advice, and Poppy Mallinson (LSHTM) for assistance with calculating the standard of living index.

Competing Interests

None declared.

Source of funding

The APCAPS household survey was funded by a Wellcome Trust Strategic Award (Grant: 084674/Z, Principal Investigator Shah Ebrahim).

Contributorship statement

The study was conceived and designed by Shah Ebrahim and VG, and overall study management was by VG, GKW, KVRK and SK. Study tools were developed by VG, GKW, and AA, and the study implemented by VG and GKW. Data management was provided by AA, GKW, SB and CB. SB was in charge of field management, SA and CB contributed to data collection and processing. RW obtained and processed the NTLI data. LO, CB, SA and SK designed the analysis reported here. LO performed the statistical analysis, SK helped interpret the results and provided crucial input on manuscript preparation. LO, CB and SK were responsible for the initial draft of the manuscript. All authors contributed to the revision of the manuscript and reviewed and approved the final version.

Data sharing

For details on how to access APCAPS data, please visit http://apcaps.lshtm.ac.uk

For peer review only

References

1 Bhutta ZA, Das JK, Rizvi A*, et al.* Evidence-based interventions for improvement of maternal and child nutrition: what can be done and at what cost? *The Lancet* 2013;**382**:452-77.

2 Haggerty PA, Rutstein SO. Breastfeeding and complementary infant feeding/postpartum effects (DHS Comparative Studies No. 30). Calverton, Maryland, USA 1999.

3 Victora CG, Bahl R, Barros AJD*, et al.* Breastfeeding in the 21st century: epidemiology, mechanisms, and lifelong effect. *The Lancet* 2016;**387**:475-90.

4 Cai X, Wardlaw T, Brown DW. Global trends in exclusive breastfeeding. *International Breastfeeding Journal* 2012;**7**:1-5.

5 Smith LC, Ruel MT, Ndiaye A. Why Is Child Malnutrition Lower in Urban Than in Rural Areas? Evidence from 36 Developing Countries. *World Development* 2005;**33**:1285-305.

6 Malhotra R, Noheria A, Amir O*, et al.* Determinants of termination of breastfeeding within the first 2 years of life in India: evidence from the National Family Health Survey-2. *Maternal and Child Nutrition* 2008;**4**:181-93.

7 Patel A, Badhoniya N, Khadse S*, et al.* Infant and young child feeding indicators and determinants of poor feeding practices in India: secondary data analysis of National Family Health Survey 2005-06. *Food & Nutrition Bulletin* 2010;**31**:314-33.

8 Chandhiok N, Singh KJ, Sahu D*, et al.* Changes in exclusive breastfeeding practices and its determinants in India, 1992–2006: analysis of national survey data. *International Breastfeeding Journal* 2015;**10**:1-13.

9 Meshram II, Laxmaiah A, Venkaiah K*, et al.* Impact of feeding and breastfeeding practices on the nutritional status of infants in a district of Andhra Pradesh, India. *National Medical Journal of India* 2012;**25**:201-6.

10 Srivastava NM, Awasthi S. Breastfeeding practices for newborns among urban poor in Lucknow, northern India: A prospective follow-up study. *Clinical Epidemiology and Global Health* 2014;**2**.

11 Noor AM, Alegana VA, Gething PW*, et al.* Using remotely sensed night-time light as a proxy for poverty in Africa. *Population Health Metrics* 2008;**6**:1-13.

12 Tatem AJ, Hay SI. Measuring urbanization pattern and extent for malaria research: a review of remote sensing approaches. *J Urban Health* 2004;**81**:363-76.

13 Dahly DL, Adair LS. Quantifying the urban environment: A scale measure of urbanicity outperforms the urban–rural dichotomy. *Social Science & Medicine* 2007;**64**:1407-19.

14 Cyril S, Oldroyd J, Renzaho A. Urbanisation, urbanicity, and health: a systematic review of the reliability and validity of urbanicity scales. *BMC Public Health* 2013;**13**:513.

15 Kinra S, Radha Krishna K, Kuper H*, et al.* Cohort Profile: Andhra Pradesh Children and Parents Study (APCAPS). *International Journal of Epidemiology* 2013;**43**:1417-24.

16 World Health Organization. Infant and young child nutrition: Global Strategy for Infant and Young Child Feeding. Geneva: World Health Organization 2003.

17 Filmer D, Pritchett L. Estimating Wealth Effects Without Expenditure Data—Or Tears: An Application To Educational Enrollments In States Of India. *Demography* 2001;**38**:115-32.

18 Vyas S, Kumaranayake L. Constructing socio-economic status indices: how to use principal components analysis. *Health Policy and Planning* 2006;**21**:459-68.

19 International Institute for Population Sciences, Mumbai. National Family Health Survey-4 (2015-16): Telegana State Factsheet. 2016 [accessed 21 April 2016]; http://rchiips.org/nfhs/pdf/NFHS4/TG_FactSheet.pdf

20 Nath DC, Goswami G. Determinants of breast-feeding patterns in an urban society of India. *Human Biology* 1997;**69**:557-73.

21 Senarath U, Agho KE, Akram DE*, et al.* Comparisons of complementary feeding indicators and associated factors in children aged 6-23 months across five South Asian countries. *Maternal & Child Nutrition* 2012;**8 (Suppl 1)**:89-106.

For peer review only - http://bmjopen.bmj.com/site/about/guidelines.xhtml

22 Senarath U, Dibley MJ, Agho KE. Factors Associated With Nonexclusive Breastfeeding in Five East and Southeast Asian Countries: A Multilevel Analysis. *Journal of Human Lactation* 2010;**26**.

23 Grummer-Strawn LM. The Effect of Changes in Population Characteristics on Breastfeeding Trends in Fifteen Developing Countries. *International Journal of Epidemiology* 1996;**25**:94-102.

24 Kapsos S, Silberman A, Bourmpoula E. Why is female labour force participation declining so sharply in India? (ILO Research Paper No.10). International Labour Office 2014.

25 Li R, Scanlon KS, Serdula MK. The Validity and Reliability of Maternal Recall of Breastfeeding Practice. *Nutrition Reviews* 2005;**63**:103-10.

26 World Health Organization. Indicators for assessing infant and young child feeding practices: Part 1 - Definitions. Geneva: WHO 2008.

27 United Nations Inter-agency Group for Child Mortality Estimation. Levels & Trends in Child Mortality: Report 2012. New York: United Nations Children's Fund 2012.

28 You D, Hug L, Ejdemyr S*, et al.* Global, regional, and national levels and trends in under-5 mortality between 1990 and 2015, with scenario-based projections to 2030: a systematic analysis by the UN Inter-agency Group for Child Mortality Estimation. *The Lancet* 2015;**386**:2275-86.

29 Sachidananda Kamath S. Reducing the Infant Mortality Rate of India to 20 by 2020: Together we can do. *Indian Pediatrics* 2015;**52**.

30 Neovita Study Group. Timing of initiation, patterns of breastfeeding, and infant survival: prospective analysis of pooled data from three randomised trials. *The Lancet Global Health* 2016;**4**:e266-e75.

31 Horta BL, Loret de Mola C, Victora CG. Long-term consequences of breastfeeding on cholesterol, obesity, systolic blood pressure and type 2 diabetes: a systematic review and meta-analysis. *Acta Paediatrica* 2015;**104**:30-7.

32 Brion M-JA, Lawlor DA, Matijasevich A*, et al.* What are the causal effects of breastfeeding on IQ, obesity and blood pressure? Evidence from comparing high-income with middle-income cohorts. *International Journal of Epidemiology* 2011.

33 Martin RM, Patel R, Kramer MS*, et al.* Effects of promoting longer-term and exclusive breastfeeding on adiposity and insulin-like growth factor-i at age 11.5 years: A randomized trial. *JAMA* 2013;**309**:1005-13.

34 Gupta A, Dadhich JP, Faridi MMA. Breastfeeding and complementary feeding as a public health intervention for child survival in India. *Indian J Pediatr* 2010;**77**:413-8.

35 Census of India 2011. Population Projections for India and States 2001-2026: Report of the Technical Group on Population Projections Constituted by the National Commission on Population. New Delhi: Office of the Registrar General and Census Commissioner 2006.

36 Renfrew MJ, McCormick FM, Wade A*, et al.* Support for healthy breastfeeding mothers with healthy term babies. *The Cochrane database of systematic reviews* 2012;**5**:CD001141-CD.

37 Rollins NC, Bhandari N, Hajeebhoy N*, et al.* Why invest, and what it will take to improve breastfeeding practices? *The Lancet* 2016;**387**:491-504.

For peer review only

For peer review only - http://bmjopen.bmj.com/site/about/guidelines.xhtml

Figure 1. Model of the association between village level urbanicity, individual level socio-economic indicators, and breastfeeding practices

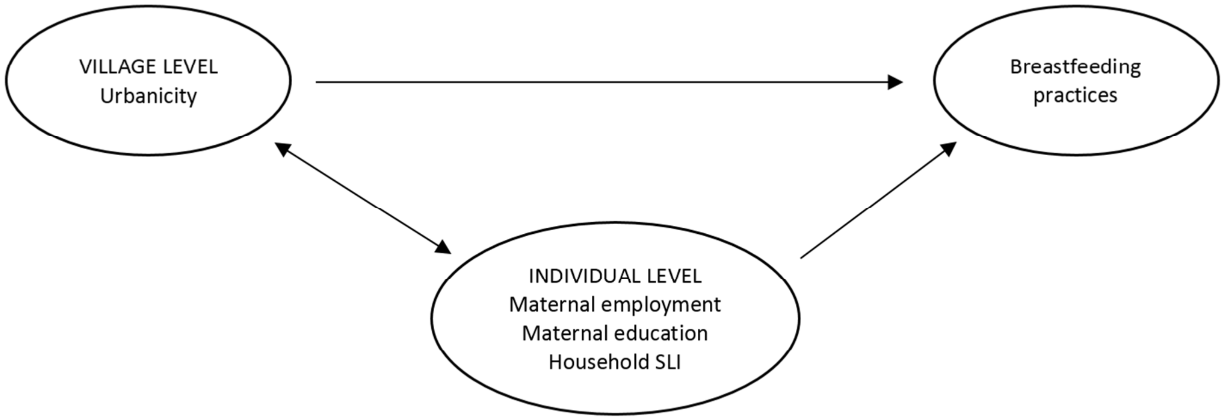

For peer review only

For peer review only - http://bmjopen.bmj.com/site/about/guidelines.xhtml

Figure 2. Flowchart of how samples for the two breastfeeding indicators were reached

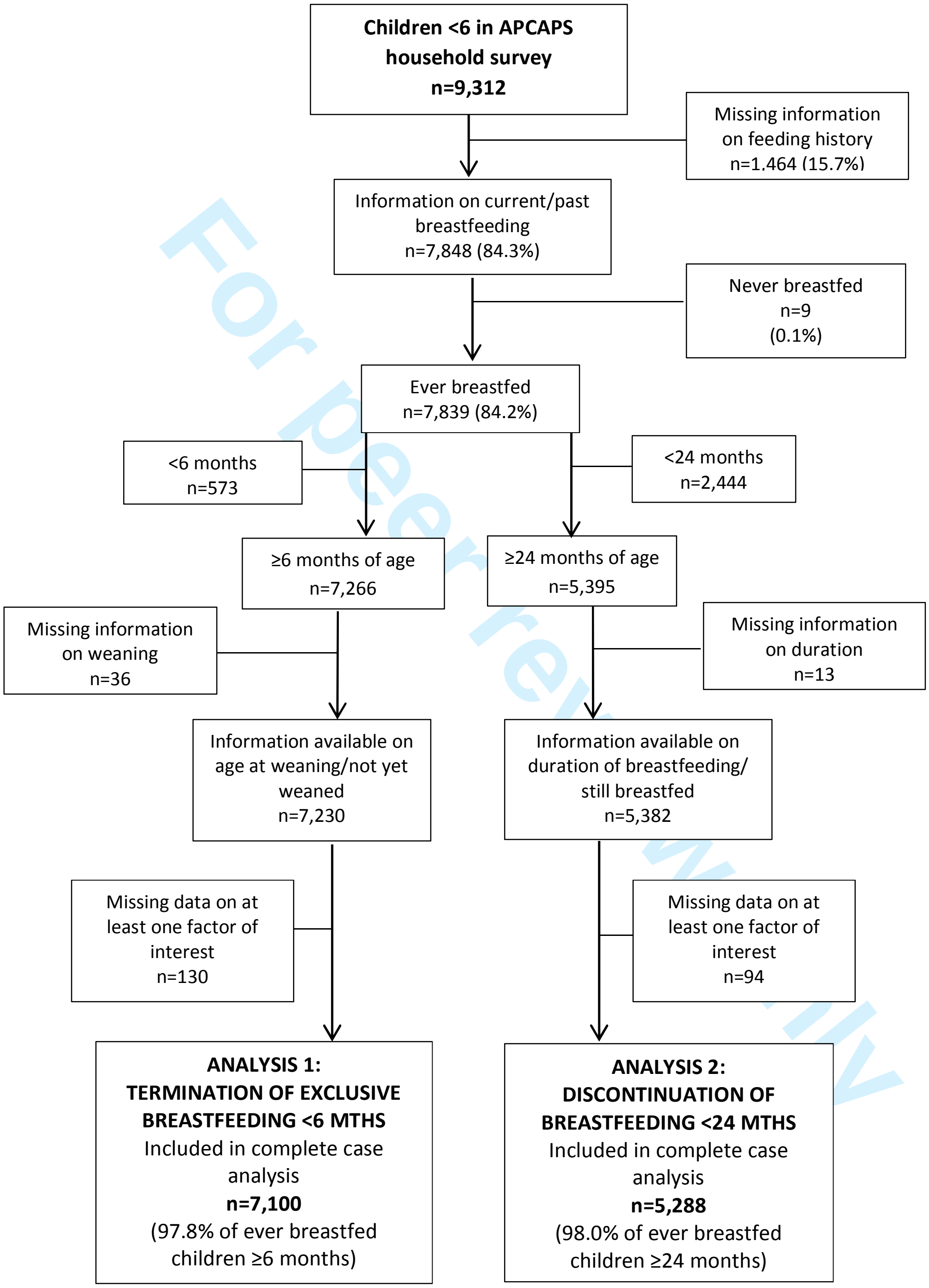

For peer review only - http://bmjopen.bmj.com/site/about/guidelines.xhtml

Table 1. Characteristics of ever breastfed children under 6 in the APCAPS household survey, by village urbanicity tertile (n=7839)

		Urbanicity (measured by night-time light intensity)						ALL	
		HIGH tertile 1 (n=4276)		MEDIUM tertile 2 (n=2082)		LOW tertile 3 (n=1476)			
		**n**	**(%)**	**n**	**(%)**	**n**	**(%)**	**n**	**(%)**
**Exclusive breastfeeding ≥6 months**[1]	Yes	2495	(63.5)	1400	(72.8)	915	(66.4)	4810	(66.5)
	No	1436	(36.5)	522	(27.2)	462	(33.6)	2420	(33.5)
**Continued breastfeeding ≥24 months**[2]	Yes	1701	(57.9)	927	(65.0)	717	(70.4)	3345	(62.2)
	No	1236	(42.1)	499	(35.0)	302	(29.6)	2037	(37.8)
**Infant sex**	Male	2189	(51.2)	1069	(51.2)	748	(50.5)	4006	(51.1)
	Female	2087	(48.8)	1013	(48.7)	733	(49.5)	3833	(48.9)
**Age of child at survey**	0-1	1337	(31.3)	650	(31.2)	457	(30.9)	2444	(31.2)
	2-3	1520	(35.5)	718	(34.5)	535	(36.1)	2773	(35.4)
	4-5	1419	(33.2)	714	(34.3)	489	(33.0)	2622	(33.4)
**Birth order**	1	1824	(43.1)	883	(42.7)	607	(41.0)	3314	(42.6)
	2	1667	(39.4)	798	(38.6)	588	(39.8)	3053	(39.2)
	≥3	743	(17.5)	389	(18.8)	284	(19.2)	1416	(18.2)
	missing	*42*		*12*		*2*		56	
	mean (SD)	1.79	(0.85)	1.80	(0.85)	1.82	(0.85)	1.80	(0.85)
**Age of mother at birth**	<20	737	(17.3)	364	(17.6)	276	(18.6)	1377	(17.6)
	20-24	2410	(56.5)	1238	(59.6)	862	(57.7)	4510	(57.7)
	25-29	917	(22.1)	393	(19.0)	288	(20.2)	1598	(20.2)
	30+	198	(44.6)	78	(3.8)	54	(3.6)	330	(4.2)
	missing	*14*		*9*		*1*		*24*	
	mean (SD)	22.9	(3.6)	22.7	(3.4)	22.7	(3.5)	22.8	(3.5)
**Family structure**	Nuclear	2866	(67.6)	1306	(63.7)	942	(64.0)	5114	(65.9)
	Joint/extended	1371	(32.4)	745	(36.3)	530	(36.0)	2646	(34.1)
	missing	*39*		*31*		*9*		79	
**Maternal education**	No formal schooling	1054	(24.7)	663	(32.0)	558	(37.7)	2275	(29.1)
	Primary	880	(20.6)	390	(18.8)	284	(19.2)	1554	(19.9)
	Secondary+	2329	(54.6)	1022	(49.3)	638	(43.1)	3989	(51.0)
	missing	*13*		*7*		*1*		*21*	
**Maternal employment**	Not working	3144	(73.8)	*1342*	(64.7)	*815*	(55.0)	5301	(67.8)
	Working	1119	(26.2)	*733*	(35.3)	*666*	(45.0)	2518	(32.2)
	missing	*13*		*7*		*0*		*20*	
**Standard of living (SLI) index**	Poorest	611	(14.3)	318	(15.3)	242	(16.3)	1171	(14.9)
	Poorer	736	(17.2)	422	(20.3)	288	(19.4)	1446	(18.5)
	Middle	816	(19.1)	458	(22.0)	378	(25.5)	1652	(21.1)
	Richer	964	(22.5)	497	(23.9)	314	(21.2)	1775	(22.6)
	Richest	1148	(26.9)	386	(18.5)	259	(17.5)	1793	(22.9)
	missing	*1*		*1*		*0*		*2*	

[1]Restricted to ever breastfed infants aged at least 6 months not yet weaned/with age at weaning (n=7230)

[2]Restricted to ever breastfed infants aged at least 24 months still breastfed/with age at cessation of breastfeeding (n=5382)

[3]chi-square test of independence for association with urbanicity

For peer review only

Table 2. Results of multilevel logistic regression models for the association between urbanicity or individual/household socio-economic factors and termination of exclusive breastfeeding <6 months, among ever-breastfed children under 6 in the APCAPS household survey[1]

				Termination of exclusive breastfeeding <6 months (n=7100)									
				**Unadjusted**		**Model 1 (null)**		**Model 2 (L1 confounders + L2 SES)**		**Model 3 (L1 confounders + L3 urbanicity)**		**Model 4 (L1 confounders + L2 SES + L3 urbanicity)**	
		**n**	**(%)**	**OR**	**(95% CI)**	**OR**	**(95% CI)**	**OR**	**(95% CI)**	**OR**	**(95% CI)**	**OR**	**(95% CI)**
FIXED EFFECTS													
**Maternal education**	No formal schooling	615	(29.1)	**ref**				ref				ref	
	Primary	534	(38.6)	3.42	(2.23, 5.25)			3.37	(2.13, 5.31)			3.36	(2.13, 5.30)
	Secondary+	1231	(38.2)	1.82	(1.29, 2.57)			1.69	(1.12, 2.54)			1.69	(1.12, 2.54)
**Maternal employment**	Not working	1584	(33.5)	ref				ref				ref	
	Working	796	(33.7)	0.94	(0.69, 1.28)			1.43	(1.00, 2.03)			1.43	(1.00-2.04)
**Standard of living (SLI) index**	Poorest	280	(26.8)	ref				ref				ref	
	Poorer	432	(32.9)	1.80	(1.07, 3.02)			1.65	(0.97, 2.81)			1.66	(0.98, 2.82)
	Middle	525	(35.1)	2.24	(1.36, 3.71)			2.08	(1.24, 3.50)			2.09	(1.24, 3.51)
	Richer	564	(35.1)	2.19	(1.33, 3.59)			1.98	(1.17, 3.34)			1.99	(1.18, 3.35)
	Richest	579	(35.3)	2.22	(1.35, 3.65)			2.11	(1.22, 3.63)			2.11	(1.23, 3.64)
	trend[2] (p value)			*0.004*				*0.003*				*0.015*	
Urbanicity	Low	459	(33.7)	ref								ref	
	Medium	517	(27.5)	0.49	(0.15, 1.56)					0.48	(0.15, 1.54)	0.48	(0.15, 1.51)
	High	1404	(36.4)	1.10	(0.35, 3.45)					1.09	(0.34, 3.43)	1.04	(0.34, 3.20)
	trend[2] (p value)			*0.87*						*0.89*		*0.91*	
RANDOM EFFECTS													
Level 2 (mothers)	*variance (SE)*					13.6194	(1.2202)	13.9036	(1.2454)	13.9888	(1.2531)	13.9072	1.2456
	PCV (compared to null)[3] (%)					ref		-2.09%		-2.71%		-2.11%	
Level 3 (villages)	*variance (SE)*					1.4949	(0.4712)	1.4429	(0.4574)	1.3870	(0.4427)	1.3200	0.4235
	PCV (compared to null)[3] (%)					ref		3.48%		7.22%		11.70%	
	PCV (compared to model 2)[4]					-		ref		-		8.52%	

[1]All ORs calculated using multilevel modelling and complete case sample (see Figure 2)
[2]Test for trend: p value including variable as linear
[3]Proportional Change in Variance ((model 1 variance – model X variance)/model 1 variance)*100%
[4]Proportional Change in Variance ((model 2 variance – model 4 variance)/model 2 variance)*100%

For peer review only - http://bmjopen.bmj.com/site/about/guidelines.xhtml

For peer review only

Table 3. Results of multilevel logistic regression models for the association between urbanicity or individual/household socio-economic factors and discontinuation of continued breastfeeding<24 months, among ever-breastfed children under 6 in the APCAPS household survey[1]

		Discontinuation of breastfeeding <24 months (n=5288)											
				Unadjusted		Model 1 (null)		Model 2 (L1 confounders + L2 SES)		Model 3 (L1 confounders + L3 urbanicity)		Model 4 (L1 confounders + L2 SES + L3 urbanicity)	
		n	(%)	OR	(95% CI)	OR	(95% CI)	OR	(95% CI)	OR	(95% CI)	OR	(95% CI)
FIXED EFFECTS													
**Maternal education**	No formal schooling	521	(30.7)	ref				ref				ref	
	Primary	395	(37.3)	1.48	(1.13, 1.94)			1.21	(0.88, 1.65)			1.21	(0.88, 1.65)
	Secondary+	1079	(42.5)	2.28	(1.82, 2.87)			1.63	(1.23, 2.16)			1.62	(1.22, 2.15)
**Maternal employment**	Not working	1384	(41.3)	ref				ref				ref	
	Working	611	(31.6)	0.55	(0.45, 0.67)			0.77	(0.60, 0.99)			0.78	(0.61, 0.99)
**Standard of (SLI) index**	Poorest	271	(34.6)	ref				ref				ref	
	Poorer	370	(37.3)	1.38	(0.99, 1.91)			1.18	(0.82, 1.69)			1.17	(0.82, 1.68)
	Middle	374	(33.1)	1.07	(0.78, 1.48)			0.86	(0.60, 1.24)			0.86	(0.60, 1.23)
	Richer	467	(39.4)	1.5	(1.09, 2.06)			1.05	(0.73, 1.51)			1.04	(0.73, 1.50)
	Richest	513	(42.9)	1.75	(1.28, 2.41)			1.09	(0.74, 1.59)			1.08	(0.74, 1.57)
	trend[2] (p value)			*0.001*				*0.027*				*0.915*	
Urbanicity	Low	290	(29.5)	ref						ref		ref	
	Medium	494	(34.6)	1.40	(0.72, 2.71)					1.45	(0.71, 2.96)	1.35	(0.66, 2.79)
	High	1213	(42.2)	2.74	(1.42, 5.28)					2.96	(1.45, 6.05)	2.64	(1.29, 5.42)
	trend[2] (p value)			*0.003*						*0.003*		*0.008*	
RANDOM EFFECTS													
Level 2 (mothers)	*variance (SE)*					3.2070	(0.4847)	4.1838	(0.6347)	4.2895	(0.6445)	4.1793	(0.6342)
	PCV (compared to null)[3] (%)					ref		-30.46%		-33.75%		-30.32%	
Level 3 (villages)	*variance (SE)*					0.6036	(0.1966)	0.6677	(0.2221)	0.4942	(0.1718)	0.5029	(0.1739)
	PCV (compared to null)[3] (%)					ref		-10.62%		18.12%		16.68%	
	PCV (compared to model 2)[4]					-		ref		-		24.68%	

[1]All ORs calculated using multilevel modelling and complete case sample (see Figure 2)
[2]Test for trend: p value including variable as linear
[3]Proportional Change in Variance ((model 1 variance – model X variance)/model 1 variance)*100%
[4]Proportional Change in Variance ((model 2 variance – model 4 variance)/model 2 variance)*100%

For peer review only

For peer review only - http://bmjopen.bmj.com/site/about/guidelines.xhtml

Supplementary table 1. Characteristics of children under 6 in the APCAPS household survey, by missing/non-missing information on breastfeeding history (n=9,312)

		Children <6 years (n=9,312) Information on current/past breastfeeding available				
		Yes (non-missing) (n=7,848)		No (missing) (n=1,464)		
		**n**	**(%)**	**n**	**(%)**	*p value*
INDIVIDUAL LEVEL						
**Infant sex**	Male	4011	(51.1)	773	(52.8)	*0.23*
	Female	3837	(48.9)	691	(47.2)	
**Age of infant**	0-1	2445	(31.2)	445	(30.4)	
	2-3	2776	(35.4)	501	(34.2)	*0.36*
	4-5	2627	(33.5)	518	(35.4)	
HOUSEHOLD LEVEL						
**Number of under 6s in household**	1	3192	(40.7)	616	(42.1)	
	2	3846	(49.0)	702	(48.0)	*0.71*
	≥3	810	(10.3)	146	(10.0)	
**Standard of living (SLI) index**	Poorest	1172	(14.9)	145	(15.5)	
	Poorer	1447	(18.4)	176	(18.8)	
	Middle	1654	(21.1)	211	(22.5)	*0.37*
	Richer	1777	(22.6)	185	(19.8)	
	Richest	1796	(22.9)	219	(23.4)	
	Missing	*2*		*528*		
VILLAGE LEVEL						
**Night-time light intensity (NTLI)**	Low	1484	(18.9)	271	(18.5)	
	Medium	2086	(26.6)	353	(24.1)	*0.09*
	High	4278	(54.5)	840	(57.4)	

For peer review only

STROBE Statement—checklist of items that should be included in reports of observational studies

	Item No	Recommendation	Location in manuscript
**Title and abstract**	1	(*a*) Indicate the study's design with a commonly used term in the title or the abstract	*In abstract (cross-sectional survey), page 2*
		(*b*) Provide in the abstract an informative and balanced summary of what was done and what was found	*Done, page 2.*
**Introduction**			
Background/rationale	2	Explain the scientific background and rationale for the investigation being reported	*Done, pages 4-5.*
Objectives	3	State specific objectives, including any prespecified hypotheses	*Done (objective in Introduction page 5, hypothesis in Methods page 8)*
**Methods**			
Study design	4	Present key elements of study design early in the paper	*Done, pages 5-6.*
Setting	5	Describe the setting, locations, and relevant dates, including periods of recruitment, exposure, follow-up, and data collection	*Done, pages 5-6.*
Participants	6	(*a*) *Cohort study*—Give the eligibility criteria, and the sources and methods of selection of participants. Describe methods of follow-up<br>*Case-control study*—Give the eligibility criteria, and the sources and methods of case ascertainment and control selection. Give the rationale for the choice of cases and controls<br>*Cross-sectional study*—Give the eligibility criteria, and the sources and methods of selection of participants	*Done, pages 5-6, 8-9 (please also see Fig 2)*
		(*b*) *Cohort study*—For matched studies, give matching criteria and number of exposed and unexposed<br>*Case-control study*—For matched studies, give matching criteria and the number of controls per case	
Variables	7	Clearly define all outcomes, exposures, predictors, potential confounders, and effect modifiers. Give diagnostic criteria, if applicable	*Done, pages 6-9.*
Data sources/ measurement	8*	For each variable of interest, give sources of data and details of methods of assessment (measurement). Describe comparability of assessment methods if there is more than one group	*Done, pages 6-8.*
Bias	9	Describe any efforts to address potential sources of bias	*Done, pages 7-8, 14-15.*
Study size	10	Explain how the study size was arrived at	*N/A (secondary analysis)*
Quantitative variables	11	Explain how quantitative variables were handled in the analyses. If applicable, describe which groupings were chosen and why	*Done, pages 6-9.*
Statistical methods	12	(*a*) Describe all statistical methods, including those used to control for confounding	*Done, pages 8-9.*
		(*b*) Describe any methods used to examine subgroups and interactions	*Done, page 9.*
		(*c*) Explain how missing data were addressed	*Done (end of para 1 'Statistical analysis' page 8, also see Supplementary Table 1)*
		(*d*) *Cohort study*—If applicable, explain how loss to follow-up was	

For peer review only - http://bmjopen.bmj.com/site/about/guidelines.xhtml

		addressed<br>*Case-control study*—If applicable, explain how matching of cases and controls was addressed<br>*Cross-sectional study*—If applicable, describe analytical methods taking account of sampling strategy	*N/A*
		(*e*) Describe any sensitivity analyses	

Continued on next page

For peer review only

Results			Location in manuscript
Participants	13*	(a) Report numbers of individuals at each stage of study—eg numbers potentially eligible, examined for eligibility, confirmed eligible, included in the study, completing follow-up, and analysed	*Done, page 9 and Figure 2.*
		(b) Give reasons for non-participation at each stage	*Done, Figure 2.*
		(c) Consider use of a flow diagram	*Provided (Figure 2).*
Descriptive data	14*	(a) Give characteristics of study participants (eg demographic, clinical, social) and information on exposures and potential confounders	*Done, Table 1.*
		(b) Indicate number of participants with missing data for each variable of interest	*Done, Table 1.*
		(c) *Cohort study*—Summarise follow-up time (eg, average and total amount)	
Outcome data	15*	*Cohort study*—Report numbers of outcome events or summary measures over time	
		Case-control study—Report numbers in each exposure category, or summary measures of exposure	
		Cross-sectional study—Report numbers of outcome events or summary measures	*Done, Table 1.*
Main results	16	(*a*) Give unadjusted estimates and, if applicable, confounder-adjusted estimates and their precision (eg, 95% confidence interval). Make clear which confounders were adjusted for and why they were included	*Done: pages 9-12, Tables 2 and 3.*
		(*b*) Report category boundaries when continuous variables were categorized	*Tables 2 and 3.*
		(*c*) If relevant, consider translating estimates of relative risk into absolute risk for a meaningful time period	
Other analyses	17	Report other analyses done—eg analyses of subgroups and interactions, and sensitivity analyses	*Done, pages 10-12.*
**Discussion**			
Key results	18	Summarise key results with reference to study objectives	*Done, pages 12-13.*
Limitations	19	Discuss limitations of the study, taking into account sources of potential bias or imprecision. Discuss both direction and magnitude of any potential bias	*Done, pages 14-15.*
Interpretation	20	Give a cautious overall interpretation of results considering objectives, limitations, multiplicity of analyses, results from similar studies, and other relevant evidence	*Done, pages 15-16.*
Generalisability	21	Discuss the generalisability (external validity) of the study results	*Done, pages 15-16.*
**Other information**			
Funding	22	Give the source of funding and the role of the funders for the present study and, if applicable, for the original study on which the present article is based	*Done, page 17.*

*Give information separately for cases and controls in case-control studies and, if applicable, for exposed and unexposed groups in cohort and cross-sectional studies.

Note: An Explanation and Elaboration article discusses each checklist item and gives methodological background and published examples of transparent reporting. The STROBE checklist is best used in conjunction with this article (freely available on the Web sites of PLoS Medicine at http://www.plosmedicine.org/, Annals of Internal Medicine at http://www.annals.org/, and Epidemiology at http://www.epidem.com/). Information on the STROBE Initiative is available at www.strobe-statement.org.

For peer review only

For peer review only - http://bmjopen.bmj.com/site/about/guidelines.xhtml

BMJ Open

Is increasing urbanicity associated with changes in breastfeeding duration in rural India? An analysis of cross-sectional household data from the Andhra Pradesh Children and Parents Study

Journal:	*BMJ Open*
Manuscript ID	bmjopen-2017-016331.R1
Article Type:	Research
Date Submitted by the Author:	08-May-2017
Complete List of Authors:	Oakley, Laura; London School of Hygiene and Tropical Medicine, Non-communicable Disease Epidemiology<br>Baker , Chris; London School of Hygiene and Tropical Medicine, Non-communicable Disease Epidemiology<br>Addanki, Srivalli; Indian Institute of Public Health<br>Gupta, Vipin; University of Delhi, Department of Anthropology<br>Walia, Gagandeep; Public Health Foundation of India, Centre for Control of Chronic Conditions<br>Aggarwal, Aastha; Public Health Foundation of India, Centre for Control of Chronic Conditions<br>Bhogadi, Santhi; Indian Institute of Public Health<br>Kulkarni, Bharati; National Institute of Nutrition<br>Wilson, Robin; University of Southampton, Geography & Environment<br>Prabhakaran, Dorairaj; Public Health Foundation of India, Centre for Control of Chronic Conditions<br>Ben-Shlomo, Yoav; University of Bristol, School of Social and Community Medicine<br>Davey Smith, George; University of Bristol, School of Social and Community Medicine<br>Radha Krishna, KV; National Institute of Nutrition<br>Kinra, Sanjay; London School of Hygiene and Tropical Medicine, Non-communicable Disease Epidemiology
**Primary Subject Heading**:	Epidemiology
Secondary Subject Heading:	Global health
Keywords:	SOCIAL MEDICINE, NUTRITION & DIETETICS, PUBLIC HEALTH

SCHOLARONE™ Manuscripts

Is increasing urbanicity associated with changes in breastfeeding duration in rural India? An analysis of cross-sectional household data from the Andhra Pradesh Children and Parents Study

Running title: Urbanicity and breastfeeding in rural India

Oakley L[1]*, Baker C[1], Addanki S[2], Gupta V[3], Walia GK[4], Aggarwal A[4], Bhogadi S[2], Kulkarni B[5], Wilson RT[6], Prabhakaran D[4], Ben-Shlomo Y[7], Smith GD[7], Radha Krishna KV[5], Kinra S[1]

[1]Department of Non-communicable Disease Epidemiology, London School of Hygiene, London, UK.

[2]Indian Institute of Public Health, Hyderabad, India.

[3]Department of Anthropology, University of Delhi, India.

[4]Centre for Control of Chronic Conditions, Public Health Foundation of India, New Delhi, India

[5]National Institute of Nutrition, Hyderabad, India.

[6]Geography & Environment, University of Southampton, Southampton, UK.

[7]School of Social and Community Medicine, University of Bristol, Bristol, UK.

*Corresponding author
Dr Laura Oakley, Assistant Professor, Department of Non-communicable Disease Epidemiology, London School of Hygiene and Tropical Medicine, Keppel St, London WC1E 7HT (laura.oakley@lshtm.ac.uk)

Main text word count: 3729 words

For peer review only - http://bmjopen.bmj.com/site/about/guidelines.xhtml

ABSTRACT

Objective: To investigate whether village-level urbanicity and lower-level socio-economic factors are associated with breastfeeding practices in transitioning rural communities in India.

Setting: 29 villages in Rangareddy district, southern India between 2011-2014.

Participants: 7,848 children under 6 years identified via a cross-sectional household survey conducted as part of the Andhra Pradesh Children and Parents Study.

Outcome measures: Two key indicators of optimal breastfeeding: termination of exclusive breastfeeding before six months and discontinuation of breastfeeding by 24 months. Village urbanicity was classified as low, medium or high according to satellite assessed night-light intensity.

Results: Breastfeeding initiation was almost universal, and approximately two in three children were exclusively breastfed to six months and a similar proportion breastfed to 24 months. Using multilevel logistic regression, increasing urbanicity was associated with breastfeeding discontinuation before 24 months (medium urbanicity OR 1.45, 95% CI 0.71-2.96; high urbanicity OR 2.96, 95% CI 1.45-6.05) but not with early (<6 months) termination of exclusive breastfeeding. Increased maternal education was independently associated with both measures of suboptimal breastfeeding, and higher household socio-economic position was associated with early termination of exclusive breastfeeding.

Conclusion: In this transitional Indian rural community, early stage urbanicity was associated with a shorter duration of breastfeeding. Closer surveillance of changes in breastfeeding practices alongside appropriate intervention strategies are recommended for emerging economies.

For peer review only

ARTICLE SUMMARY

- Previous studies have investigated the association between urbanisation and breastfeeding using the urban-rural dichotomy.
- We used data from a large rural cohort in southern India that is currently undergoing rapid and uneven urbanisation due to its proximity to a major urban centre.
- The use of night-time light intensity data as an indicator of urbanicity allowed us to examine subtler changes in breastfeeding practices along the urban-rural continuum.
- Sixteen percent of children were excluded from the analysis due to missing information on breastfeeding practices.
- We relied on maternal retrospective recall of breastfeeding events for our outcome measurement.

For peer review only

For peer review only - http://bmjopen.bmj.com/site/about/guidelines.xhtml

INTRODUCTION

The promotion of breastfeeding is one of the three interventions identified as having the largest potential impact on global child death[1]. Optimal breastfeeding is defined by the World Health Organisation (WHO) as early breastfeeding initiation, exclusive breastfeeding to six months, and continued breastfeeding to two years or beyond alongside appropriate complementary feeding. Many low and middle income countries (LMICs) have a strong tradition of near universal and prolonged breastfeeding[2,3], though exclusive breastfeeding to six months (hereafter referred to simply as 'exclusive breastfeeding') is less common. A small increase in the global proportion of children exclusively breastfed between 1995 and 2010 has been reported, but the overall proportion (40%) still falls strikingly short of universal coverage and obscures differences in country-specific trends[4].

Many LMICs are currently experiencing a rapid increase in the proportion of people living in built-up areas, and the social, cultural and economic changes associated with this process of urbanisation have the potential to impact on traditional breastfeeding practices. Direct threats to optimal breastfeeding include early introduction of other liquids, and inappropriate supplementation with solid or semi-solid foods. These behaviours may be influenced by changing social norms, for example increasing numbers of mothers working outside the home. Of all positive health behaviours, breastfeeding is one of the few more prevalent in LMICs compared to HICs[3]. Within LMICs this trend is mirrored by a higher prevalence of suboptimal breastfeeding in urban areas compared to rural areas[5]: a trend also observed in India[6-8] alongside variation by various socio-economic indicators[6-10]. Although the high level urban-rural comparison is of interest, there may also be subtler changes in breastfeeding practices along the urban-rural continuum given the peri-urban effects on villages close to urban centres. These changes can potentially be investigated by using a measure of 'urbanicity' which aims to assess the extent of urbanisation in a given area. A number of different indicators of urbanicity have evolved, including the use of remote light sensing[11,12], and multi-component scales[13,14]. The early identification of changes in breastfeeding practices

For peer review only - http://bmjopen.bmj.com/site/about/guidelines.xhtml

accompanying the urbanicity transition - and an understanding of the underlying mechanisms - are necessary for informing appropriate interventions to protect traditionally positive breastfeeding practices in transitioning communities.

The Andhra Pradesh Children and Parents Study (APCAPS) is a rural socio-demographic cohort in southern India that is currently undergoing rapid and uneven urbanisation due to its proximity to a major urban centre (Hyderabad), providing a unique opportunity to examine the association between early stage urbanicity and breastfeeding practices.

METHODS

Study design

APCAPS is an intergenerational cohort originally established to study the long-term effects of early-life undernutrition on risk of cardiovascular disease and subsequently expanded to include trans-generational influences of other environmental and genetic factors on chronic diseases in transitioning rural India.

The original cohort is based on the participants in the Hyderabad Nutrition Trial (HNT) conducted in 1987–90 in 29 villages approximately 50-100km from Hyderabad in Telangana state (formally Andhra Pradesh), southern India[15]. The dataset used in this analysis is based on a cross-sectional household survey conducted between 2011 and 2014 in the study villages. All households (household defined as a group of people living in the same residence and sharing a common kitchen) in the study villages were visited by fieldworkers and socio-demographic information was collected on each household. In addition, a basic health profile was collected for each child under 6 years of age, comprising information on infant feeding (colostrum intake, total duration of breastfeeding, age of onset of weaning), immunization and anthropometric measurements. Fieldworkers made repeated visits to households to maximise response and to clarify inconsistencies in collected data. Data were

For peer review only - http://bmjopen.bmj.com/site/about/guidelines.xhtml

collected from 23,314 households in total, of which 5,968 (25.6%) included at least one child under 6 years.

The study received approval from the ethics committees of the National Institute of Nutrition (NIN) (Hyderabad, India) and London School of Hygiene and Tropical Medicine (London, UK). Approval was also sought from the Indian Council for Medical Research and the village heads and their committees in each of the study villages. Written informed consent (or witnessed thumbprint if illiterate) was obtained from the participants prior to their inclusion in the study.

Breastfeeding outcomes and explanatory variables

Two breastfeeding outcomes were used in this analysis: termination of exclusive breastfeeding (EBF) before six months, and discontinuation of breastfeeding before 24 months. These outcomes reflect failure to achieve two of the specific WHO recommendations for optimum feeding practices (exclusive breastfeeding to six months and continued breastfeeding to two years)[16]. As part of the basic health profile for children compiled for children under 6 in the household survey, mothers were asked to report the total duration of breastfeeding (in months), and the age (in months) at onset of weaning. 'Weaning' was defined by fieldworkers as the age at which the child was given anything other than mother's milk, i.e. age at initiation of complementary feeding. A copy of the questions used in the survey is provided as a supplementary figure (figure S1).

Our primary explanatory factor was urbanicity, measured using remotely-sensed village-level night-time light intensity (NTLI) scores, as these are objective, unbiased and easily available over wide areas. Although this analysis represents the first application of NTLI data to the APCAPS population, NTLI data is increasingly being used as an area-based indicator of socio-economic development[11,12]. The light which is included in the NTLI score include any outside lights, ranging from fires and gas flares to lights related to human settlements. Low level lights such as from streets

For peer review only - http://bmjopen.bmj.com/site/about/guidelines.xhtml

and car headlights can be observed if there is a sufficient number of sources, but indoor lights cannot be observed. NTLI scores were calculated for 2012 using the National Oceanic and Atmospheric Administration (NOAA) Stable Lights product which provides yearly average night-time light intensity measures processed and filtered to remove events such as fires and lightening contamination by cloud or moon reflections and background noise, at a 1km resolution. Scores for each village were calculated by summing the raw NTLI values over each village polygon (digitised using Bing Maps combined with GPS-based surveying by the field teams). The 1km-resolution NTLI data was upscaled to 100m resolution to allow more accurate estimation of the NTLI values covered by each polygon, as many villages are small and partially cover multiple 1km grid cells.

NTLI scores for each village were validated against alternative urbanicity measurements (field worker ranking and a multi-component urbanicity score based on household-level material assets and village-level availability of infrastructure and services) showing positive correlations (0.65 and 0.53 respectively) . Study villages were ranked by their NTLI score and divided into tertiles to represent 'low' (10 villages), 'medium' (10 villages) and 'high' (9 villages) levels of urbanicity. The NTLI tertile scores matched the field worker ranking in 50% of the villages, and cases of disagreement between NTLI and field worker ranking, the latter was more conservative and ranked villages as medium urbanicity rather than high urbanicity. Only one village had a significant divergence between NTLI and fieldworker ranking.

In addition, we investigated mother-level socio-economic factors which may be correlated with urbanicity: maternal education (no formal education, primary education, or secondary education and higher), maternal employment (paid work vs. no paid work), and a household level standard of living index (SLI). Asset-based SLIs have been established as a valid proxy measure of household wealth[17]. We generated a SLI score for each household, calculated by using information on household assets including house and land ownership, characteristics of the home (electricity, water pump, separate kitchen, separate toilet) and ownership of various assets (tractor, radio, AC, washing machine, bore

For peer review only - http://bmjopen.bmj.com/site/about/guidelines.xhtml

hole, telephone, TV, fridge, bicycle, two wheeler, four wheeler, bank account, animal cart, sofa, cot/bed, mattress, table). Principal component analysis (PCA) was used to determine the weights for each component in the index[18], and households were divided into quintiles according to their weighted score. We also report data on a number of other factors likely to be associated with breastfeeding practices: sex of child, birth order, maternal age (grouped), and household composition (joint/extended or nuclear).

Statistical analysis

We included in the analysis all children under 6 years who were breastfed at least once and for whom information was available on feeding history. The analysis investigating termination of EBF before six months was restricted to children six months or older at the time of survey, and correspondingly only those children aged 24 months or older were included in the analysis of discontinuation of breastfeeding before 24 months. A small proportion of children (3%) had missing information on one or more variables of interest and were excluded.

We hypothesised that urbanicity would be associated with less favourable breastfeeding practices. This could operate through at least two different indirect pathways (see figure 1): through increasing individual-level employment, education or assets so that households are less likely to maintain breastfeeding, or due to more urbanised villages have a different 'collective' attitude to breastfeeding. We investigated these hypotheses by using multilevel logistic regression modelling with children (level 1) nested within mothers (level 2, max n=5,477) nested within villages (level 3, n=29). This approach allowed us to model the variation in breastfeeding outcomes at each level (random effects), and to estimate the effect of specific mother and village-level factors on breastfeeding practices (fixed effects). We initially fitted a null model (model 1) for each of the two outcomes with random intercepts only in order to estimate the baseline between-mother and

For peer review only - http://bmjopen.bmj.com/site/about/guidelines.xhtml

between-village variance. We then fitted a series of models for each breastfeeding outcome, adding covariates as fixed effects to the included random effects, where fixed effects were interpreted as the average effect on the specified breastfeeding outcome across all mothers and villages. These models included individual demographic factors and mother-level socioeconomic indicators (model 2), individual demographic factors and village-level urbanicity (model 3), and all variables (model 4). Due to the correlation between socio-economic indicators and urbanicity, we considered estimates from model 3 our main results. Proportional change in variance (PCV) was calculated as a measure of change in mother-level (level 2) and village-level (level 3) variance between the null model and subsequent models, and (for village-level variance only) the measure of change between a model with (model 4) and without (model 2) the village-level urbanicity variable included.

Estimates of the association between mother-level socio-economic variables and breastfeeding outcomes were derived from model 2 (adjusted for individual-level demographic variables, but not urbanicity).

We hypothesised *a priori* that the association between village-level urbanicity and breastfeeding may vary by household SLI and maternal education. We investigated these cross-level interactions in further models (for SLI, comparing the richest two quintiles to the three poorest quintiles; for education, comparing secondary education versus no or primary education).

All statistical analyses were conducted using Stata 14 (StataCorp, College Station, Texas, USA).

RESULTS

Characteristics of the sample

Information on breastfeeding was available on a total of 7,848 children (5,390 households), 99% (n=7,839) of whom were breastfed at least once (figure 2).

For peer review only - http://bmjopen.bmj.com/site/about/guidelines.xhtml

The characteristics of ever breastfed children by urbanicity of village are presented in table 1. There was little variation in infant sex, birth order or maternal age by urbanicity of village. Children residing in villages classified as more urbanised had mothers that were more likely to have been educated to secondary level, less likely to have mothers in paid employment, and a higher standard of living. Joint/extended families were slightly less prevalent in high urbanicity villages.

Termination of EBF by six months

Among the 7,142 children no longer exclusively breastfed (88 children ≥6 months were still exclusively breastfed at the time of survey), the mean age at termination of EBF was 6.1 months (sd 1.8), median 6.0 months, and intra-quartile (IQ) range 5-6 months. One third of children (33.5%, n=2,420) were EBF for a period of less than six months (table 1).

Fixed effects

There was no statistically significant trend regarding early termination of EBF and village level urbanicity. The prevalence of early termination of EBF was lowest in medium urbanicity villages (27.2%), higher in lower urbanicity villages (33.6%), and highest in high urbanicity villages (36.5%). In multivariable analysis there was no evidence that urbanicity was associated with termination of EBF by six months (model 3, table 2), with little change in estimates after the addition of demographic and socio-economic covariates to the model.

After adjustment for other individual- and mother-level covariates, both children of mothers with primary education and children of mothers with secondary education were more likely to be EBF for less than six months when compared to children of mothers with no formal education (primary education OR 3.37, 95% CI 2.13-5.31; secondary education OR 1.69, 95% CI 1.12-2.54; model 2, table 2). Increasing SLI quintile was associated with up to twice the odds of early termination of EBF compared to children from the poorest households (richest quintile OR 2.11, 95% CI 1.22-3.63; p

For peer review only - http://bmjopen.bmj.com/site/about/guidelines.xhtml

value for trend = 0.003). There was some evidence that maternal employment was also associated with early termination of EBF (OR 1.43, 95% CI 1.00-2.03). The estimates for socio-economic variables did not change with the addition of urbanicity to the model.

Interaction effects

There was no evidence of interaction between urbanicity and either household SLI or maternal education.

Random effects

There was statistically significant unexplained variance estimates at both the mother and village level (model 1, table 1). Unexplained variability was consistently higher at the mother level compared to the village level. The addition of individual level parameters resulted in a slight decline in community-level variation (variance 1.50 and 1.44 in models 1 and 2 respectively). There was a further decline in village-level variance when the urbanicity variable was added to the model (variance 1.32 in model 4). Comparing the village-level variance between model 2 and model 4 suggests that 8.5% of the observed village-level variation can be explained by urbanicity.

Discontinuation of breastfeeding by 24 months

At the time of survey, 784 children aged ≥24 months were still being breastfed. Among those children no longer breastfed, the mean and median age at discontinuation of breastfeeding was 21.4 (sd 8.5) and 24 months respectively, and the IQR was 15-24 months. Nearly four in ten children (37.8%, n=2037) were breastfed for less than 24 months in total.

Fixed effects

Discontinuation of breastfeeding by 24 months was more common in high urbanicity villages (42.1%) and least common in low urbanicity villages (29.6%). After adjustment for individual-level

For peer review only - http://bmjopen.bmj.com/site/about/guidelines.xhtml

demographic factors, high urbanicity was associated with increased odds of breastfeeding discontinuation before 24 months (OR 2.64, 95% CI 1.29, 5.42; model 3, table 3). The OR for medium urbanicity was slightly increased, though not statistically significant at $p < 0.05$ (OR 1.45, 95% CI 0.71, 2.96; model 4) and there was evidence of a linear trend (p value 0.008). Additional adjustment for socio-economic variables resulted in a slight reduction in the odds ratios (high urbanicity OR 2.64, 95% CI 1.29, 5.42; medium urbanicity OR 1.35, 95% CI 0.66, 2.79; model 4).

When compared to children of mothers with no formal schooling, children of mothers with secondary education were at significantly higher odds of breastfeeding discontinuation after adjustment for all demographic and socio-economic factors (OR 1.63, 95% CI 1.23-2.16; model 2, table 3). Maternal employment was associated with a slight reduction in the odds of breastfeeding discontinuation before 24 months (OR 0.77, 95% CI 0.60-0.99). There was no evidence that SLI quintile was independently associated with breastfeeding discontinuation by 24 months. The inclusion of urbanicity in the model did not alter the socio-economic estimates of effect.

Interaction effects

There was no evidence of interaction between urbanicity and mother-level socio-economic factors (household SLI and maternal education).

Random effects

The random effects parameters for models investigating discontinuation of breastfeeding before 24 months are presented in table 3. In the null model (model 1) the proportion of residual variance attributable to mothers (level 2, 53.7%) was much higher than the variance attributable to villages (level 3, 8.5%). The addition of urbanicity to a model including individual and mother-level factors resulted in a decrease of 8.5% in village-level variance.

For peer review only - http://bmjopen.bmj.com/site/about/guidelines.xhtml

DISCUSSION

Summary of main findings

In this study approximately two in three children were exclusively breastfed to six months and a similar proportion breastfed to 24 months. At the village level, high urbanicity was associated with breastfeeding discontinuation before 24 months, but there was no evidence that urbanicity was associated with early termination of EBF. At the mother level, increased maternal education was independently associated with both indicators of suboptimal breastfeeding, and high SLI associated with an increased odds of EBF for less than 6 months. Maternal employment showed a variable association with breastfeeding. The residual variation in breastfeeding outcomes suggested greater heterogeneity within-villages than between-villages.

Consistency with previous studies

Our estimates of breastfeeding prevalence are largely consistent with those derived from other population-based studies in India. Early results from NFHS-4 (2015-16) Telangana state indicate that 67.3% of infants aged 0-6 months (at the time of survey) were exclusively breastfed[19], and a study of 600 mother-child pairs in Andhra Pradesh reports that 75% of infants aged 3-5 months were exclusively breastfed[20]. Some of the younger infants included in these two study samples will have ceased breastfeeding by six months, suggesting that our study sample has a slightly higher proportion of exclusive breastfeeding to six months. The overall proportion of children breastfed until at least 24 months in our study was almost identical to an analysis of all-India NFHS-2 data: (62.2% vs. 63%) [6].

Very few existing studies have investigated the association between urbanicity and breastfeeding. In one study based in the Philippines, Dahly et al. reported that length of breastfeeding was negatively correlated with increasing urbanicity (using a multicomponent measure)[13]. The persisting association between high urbanicity and increased odds of breastfeeding discontinuation<24 months - after

For peer review only - http://bmjopen.bmj.com/site/about/guidelines.xhtml

adjustment for lower level socio-economic circumstances - reported in our study support the findings from Dahly et al.

Increasing urbanicity is associated with positive socio-economic changes such as improved education for women and increased income and household wealth. A number of other studies from India and other LMICs have demonstrated a negative association between improved socio-economic position and breastfeeding practices[6-8,21-24]. We found similar results with regard to household SLI and increased maternal education, and early termination of exclusive breastfeeding. One explanation for this trend could be the greater affordability and/or social desirability of commercial breast milk substitutes. The association between education and early termination of exclusive breastfeeding was strongest for primary education (primary education OR 3.37, 95% CI 2.13-5.31; secondary education OR 1.69, 95% CI 1.12-2.54). This suggests that while education in general is associated with a reduction in the length of exclusive breastfeeding, higher levels of education partially ameliorate this effect. Interestingly, there was some evidence that maternal employment had a protective effect on breastfeeding discontinuation by 24 months, though the opposite trend was observed with regard to early cessation of EBF. There is some evidence of a U-shaped association between education and women's employment in India, with paid employment outside the home common among women with little or no formal education, lower among women with moderate levels of education, and rising again with high levels of education[25]. Mothers in employment are likely to be a heterogeneous group, making it difficult to draw any firm conclusions about the association between paid employment and breastfeeding practices in this sample.

Strengths and limitations

The APCAPS cohort provides a unique opportunity to investigate current health behaviour and outcomes set against the backdrop of rapid urbanisation and economic transition in rural India. While

For peer review only - http://bmjopen.bmj.com/site/about/guidelines.xhtml

studies based on the high level urban-rural comparison may help to predict the impact of 'total' urbanisation on breastfeeding practices, they obscure the temporal emergence of subtler changes in the urban environment which may be amenable to intervention. The use of multilevel models enabled us to explore the role of factors at different levels: individual, mother, and village.

Although the vast majority of all under 6s in the study villages were included in our analysis, 15.7% (n=1,464) were excluded due to missing information on feeding history due to the mother living elsewhere, travelling, or deceased. In a comparison of included and excluded children there was no evidence that infant sex, infant age, number of under 6s living in the household, or household SLI differed by missing status (supplementary table 1). A slightly higher proportion of excluded children resided in high urbanicity villages (p 0.09).

We relied on maternal recall of breastfeeding events for our outcome measurement. For the analysis of exclusive breastfeeding at six months, the recall period ranged from 0 to 5.5 years, and for breastfeeding continuation at 24 months the recall period was 0 to 4 years. A review of 11 studies assessing the validity and reliability of maternal recall of breastfeeding concluded that maternal recall of breastfeeding duration is good, especially when the recall period is short (<3 years)[26]. A more recent study, conducted in a population where breastfeeding initiation was near universal and duration long, found that even after twenty years, 64% of women recalled duration correctly to within one month (90% within three months)[27]. However, there is some evidence that recall of age at introduction of complementary foods or non-breastmilk fluids is less accurate[26]. It is unclear whether any misclassification of breastfeeding behaviour is independent of other characteristics, but where differential misclassification has been suggested, more highly educated or wealthier mothers have tended to over-report breastfeeding[28]. Given that these characteristics were associated with suboptimal breastfeeding practices in this study, we may have underestimated any true difference in breastfeeding by socio-demographic characteristics.

For peer review only - http://bmjopen.bmj.com/site/about/guidelines.xhtml

Our measure of urbanicity was derived from night-time light intensity data, information which is objective, regularly updated and free to use. Additionally, data on night-time light intensity is available over a number of years and could be used in future studies to investigate trends in urbanicity over time. However, it must be noted that urbanicity is an ecological indicator and as such may not accurately reflect individual environment, particularly given that many women may travel regularly outside their home village for work or family reasons.

Implications

Nearly a quarter (24%) of all global under-five deaths occur in India[29]. In light of the failure to achieve the Millennium Development Goal infant mortality rate (IMR) target reduction[30], a new target of reducing the IMR to 20 per 1,000 live births by 2020 has recently been proposed[31]. Early results from the latest National Family Health Survey (NFHS-4) data collected in Telegana state report a current IMR of 28 (20 in urban areas, 35 in rural areas)[19]. An increase in optimal breastfeeding practices will help to achieve improvements in infant survival, in addition to reducing the considerable burden of infant morbidity[3,32]. India faces an ever-increasing epidemic of chronic disease in common with many other LMICs. Several studies have suggested that breastfeeding has a protective effect on long-term outcomes such as obesity and diabetes in adulthood[33], though residual confounding is difficult to exclude[34], and the most recent data from the PROBIT RCT do not support an association between breastfeeding and adiposity in late childhood[35].

A substantial proportion of infants in India are exclusively breastfed for less than the six months recommended by WHO[7,9,10], and a recent study reported that there was little change in the prevalence of exclusive breastfeeding in India between 1992-1993 and 2005-2006[8] . The lack of country-specific holistic and coordinated policy programmes supporting breastfeeding has also been highlighted[36].

For peer review only - http://bmjopen.bmj.com/site/about/guidelines.xhtml

Therefore, research to further understand the determinants of suboptimal breastfeeding practices in India is timely.

Our findings suggest that in LMICs with a strong tradition of breastfeeding, negative changes in breastfeeding behaviour may be observed during early stages of the urbanicity transition. Reduced duration of breastfeeding among more educated mothers may be one of the earliest markers of this change. India is currently undergoing rapid urbanisation, with the proportion of the population living in towns and cities is set to increase from an estimated 28% in 2011 to 38% by 2026[37]. Many more individuals live in areas which though traditionally described as rural are increasingly displaying many of the characteristics of urban areas. There is good evidence that breastfeeding behaviours are amenable to change through interventions delivered at the household and community level, as well as those targeting health systems[38,39]. Intervention programmes to protect and promote breastfeeding should be considered in transitioning communities to counteract changes in breastfeeding practices, preferably targeted at those mothers identified as most at risk of suboptimal breastfeeding practices.

For peer review only

For peer review only - http://bmjopen.bmj.com/site/about/guidelines.xhtml

Acknowledgements

We wish to acknowledge our dedicated field teams led by Santhi Bhogadi and the study participants who made this study possible. We also acknowledge the contribution of Naveen Chittaluri and Ekta Jain to data processing and management. We also thank Cono Ariti at the London School of Hygiene and Tropical Medicine (LSHTM) who provided statistical advice, and Poppy Mallinson (LSHTM) for assistance with calculating the standard of living index.

Competing Interests

None declared.

Source of funding

The APCAPS household survey was funded by a Wellcome Trust Strategic Award (Grant: 084674/Z, Principal Investigator Shah Ebrahim).

Contributorship statement

The study was conceived and designed by Shah Ebrahim and VG, and overall study management was by VG, GKW, KVRK and SK. Study tools were developed by VG, GKW, and AA, and the study implemented by VG and GKW. Data management was provided by AA, GKW, SB and CB. SB was in charge of field management, SA and CB contributed to data collection and processing. RW obtained and processed the NTLI data. LO, CB, SA and SK designed the analysis reported here. LO performed the statistical analysis, SK helped interpret the results and provided crucial input on manuscript preparation. LO, CB and SK were responsible for the initial draft of the manuscript. All authors contributed to the revision of the manuscript and reviewed and approved the final version.

Data sharing

For details on how to access APCAPS data, please visit http://apcaps.lshtm.ac.uk

For peer review only

For peer review only - http://bmjopen.bmj.com/site/about/guidelines.xhtml

References

1. Bhutta ZA, Das JK, Rizvi A, et al. Evidence-based interventions for improvement of maternal and child nutrition: what can be done and at what cost? The Lancet 2013;**382**(9890):452-77 doi: http://dx.doi.org/10.1016/S0140-6736(13)60996-4[published Online First: Epub Date]|.
2. Haggerty PA, Rutstein SO. Breastfeeding and complementary infant feeding/postpartum effects (DHS Comparative Studies No. 30). Calverton, Maryland, USA, 1999.
3. Victora CG, Bahl R, Barros AJD, et al. Breastfeeding in the 21st century: epidemiology, mechanisms, and lifelong effect. The Lancet 2016;**387**(10017):475-90 doi: 10.1016/S0140-6736(15)01024-7[published Online First: Epub Date]|.
4. Cai X, Wardlaw T, Brown DW. Global trends in exclusive breastfeeding. International Breastfeeding Journal 2012;**7**(1):1-5 doi: 10.1186/1746-4358-7-12[published Online First: Epub Date]|.
5. Smith LC, Ruel MT, Ndiaye A. Why Is Child Malnutrition Lower in Urban Than in Rural Areas? Evidence from 36 Developing Countries. World Development 2005;**33**(8):1285-305 doi: http://dx.doi.org/10.1016/j.worlddev.2005.03.002[published Online First: Epub Date]|.
6. Malhotra R, Noheria A, Amir O, et al. Determinants of termination of breastfeeding within the first 2 years of life in India: evidence from the National Family Health Survey-2. Maternal and Child Nutrition 2008;**4**(3):181-93
7. Patel A, Badhoniya N, Khadse S, et al. Infant and young child feeding indicators and determinants of poor feeding practices in India: secondary data analysis of National Family Health Survey 2005-06. Food & Nutrition Bulletin 2010;**31**(2):314-33
8. Chandhiok N, Singh KJ, Sahu D, et al. Changes in exclusive breastfeeding practices and its determinants in India, 1992–2006: analysis of national survey data. International Breastfeeding Journal 2015;**10**(1):1-13 doi: 10.1186/s13006-015-0059-0[published Online First: Epub Date]|.
9. Meshram II, Laxmaiah A, Venkaiah K, et al. Impact of feeding and breastfeeding practices on the nutritional status of infants in a district of Andhra Pradesh, India. National Medical Journal of India 2012;**25**(4):201-06
10. Srivastava NM, Awasthi S. Breastfeeding practices for newborns among urban poor in Lucknow, northern India: A prospective follow-up study. Clinical Epidemiology and Global Health 2014;**2**(2)
11. Noor AM, Alegana VA, Gething PW, et al. Using remotely sensed night-time light as a proxy for poverty in Africa. Population Health Metrics 2008;**6**(1):1-13 doi: 10.1186/1478-7954-6-5[published Online First: Epub Date]|.
12. Tatem AJ, Hay SI. Measuring urbanization pattern and extent for malaria research: a review of remote sensing approaches. J Urban Health 2004;**81**(3):363-76
13. Dahly DL, Adair LS. Quantifying the urban environment: A scale measure of urbanicity outperforms the urban–rural dichotomy. Social Science & Medicine 2007;**64**(7):1407-19 doi: http://dx.doi.org/10.1016/j.socscimed.2006.11.019[published Online First: Epub Date]|.
14. Cyril S, Oldroyd J, Renzaho A. Urbanisation, urbanicity, and health: a systematic review of the reliability and validity of urbanicity scales. BMC Public Health 2013;**13**(1):513
15. Kinra S, Radha Krishna K, Kuper H, et al. Cohort Profile: Andhra Pradesh Children and Parents Study (APCAPS). International Journal of Epidemiology 2013;**43**(5):1417-24 doi: 10.1093/ije/dyt128[published Online First: Epub Date]|.
16. World Health Organization. Infant and young child nutrition: Global Strategy for Infant and Young Child Feeding. Geneva: World Health Organization, 2003.
17. Filmer D, Pritchett L. Estimating Wealth Effects Without Expenditure Data—Or Tears: An Application To Educational Enrollments In States Of India. Demography 2001;**38**(1):115-32 doi: 10.1353/dem.2001.0003[published Online First: Epub Date]|.
18. Vyas S, Kumaranayake L. Constructing socio-economic status indices: how to use principal components analysis. Health Policy and Planning 2006;**21**(6):459-68 doi: 10.1093/heapol/czl029[published Online First: Epub Date]|.

For peer review only

19. International Institute for Population Sciences, Mumbai. National Family Health Survey-4 (2015-16): Telegana State Factsheet. Secondary National Family Health Survey-4 (2015-16): Telegana State Factsheet 2016. http://rchiips.org/nfhs/pdf/NFHS4/TG_FactSheet.pdf.
20. Shroff MR, Griffiths PL, Suchindran C, et al. Does maternal autonomy influence feeding practices and infant growth in rural India? Social science & medicine (1982) 2011;**73**(3):447-55 doi: 10.1016/j.socscimed.2011.05.040[published Online First: Epub Date]|.
21. Nath DC, Goswami G. Determinants of breast-feeding patterns in an urban society of India. Human Biology 1997;**69**(4):557-73
22. Senarath U, Agho KE, Akram DE, et al. Comparisons of complementary feeding indicators and associated factors in children aged 6-23 months across five South Asian countries. Maternal & Child Nutrition 2012;**8 (Suppl 1)**:89-106
23. Senarath U, Dibley MJ, Agho KE. Factors Associated With Nonexclusive Breastfeeding in Five East and Southeast Asian Countries: A Multilevel Analysis. Journal of Human Lactation 2010;**26**(3) doi: 10.1177/0890334409357562[published Online First: Epub Date]|.
24. Grummer-Strawn LM. The Effect of Changes in Population Characteristics on Breastfeeding Trends in Fifteen Developing Countries. International Journal of Epidemiology 1996;**25**(1):94-102 doi: 10.1093/ije/25.1.94[published Online First: Epub Date]|.
25. Kapsos S, Silberman A, Bourmpoula E. Why is female labour force participation declining so sharply in India? (ILO Research Paper No.10): International Labour Office, 2014.
26. Li R, Scanlon KS, Serdula MK. The Validity and Reliability of Maternal Recall of Breastfeeding Practice. Nutrition Reviews 2005;**63**(4):103-10 doi: 10.1111/j.1753-4887.2005.tb00128.x[published Online First: Epub Date]|.
27. Natland ST, Andersen LF, Nilsen TIL, et al. Maternal recall of breastfeeding duration twenty years after delivery. BMC Medical Research Methodology 2012;**12**(1):179 doi: 10.1186/1471-2288-12-179[published Online First: Epub Date]|.
28. Huttly SRA, Barros FC, Victora CG, et al. DO MOTHERS OVERESTIMATE BREAST FEEDING DURATION? AN EXAMPLE OF RECALL BIAS FROM A STUDY IN SOUTHERN BRAZIL. American Journal of Epidemiology 1990;**132**(3):572-75 doi: 10.1093/oxfordjournals.aje.a115693[published Online First: Epub Date]|.
29. United Nations Inter-agency Group for Child Mortality Estimation. Levels & Trends in Child Mortality: Report 2012. New York: United Nations Children's Fund, 2012.
30. You D, Hug L, Ejdemyr S, et al. Global, regional, and national levels and trends in under-5 mortality between 1990 and 2015, with scenario-based projections to 2030: a systematic analysis by the UN Inter-agency Group for Child Mortality Estimation. The Lancet 2015;**386**(10010):2275-86 doi: http://dx.doi.org/10.1016/S0140-6736(15)00120-8[published Online First: Epub Date]|.
31. Sachidananda Kamath S. Reducing the Infant Mortality Rate of India to 20 by 2020: Together we can do. Indian Pediatrics 2015;**52**(194)
32. Neovita Study Group. Timing of initiation, patterns of breastfeeding, and infant survival: prospective analysis of pooled data from three randomised trials. The Lancet Global Health 2016;**4**(4):e266-e75 doi: http://dx.doi.org/10.1016/S2214-109X(16)00040-1[published Online First: Epub Date]|.
33. Horta BL, Loret de Mola C, Victora CG. Long-term consequences of breastfeeding on cholesterol, obesity, systolic blood pressure and type 2 diabetes: a systematic review and meta-analysis. Acta Paediatrica 2015;**104**:30-37 doi: 10.1111/apa.13133[published Online First: Epub Date]|.
34. Brion M-JA, Lawlor DA, Matijasevich A, et al. What are the causal effects of breastfeeding on IQ, obesity and blood pressure? Evidence from comparing high-income with middle-income cohorts. International Journal of Epidemiology 2011 doi: 10.1093/ije/dyr020[published Online First: Epub Date]|.
35. Martin RM, Patel R, Kramer MS, et al. Effects of promoting longer-term and exclusive breastfeeding on adiposity and insulin-like growth factor-i at age 11.5 years: A randomized trial. JAMA 2013;**309**(10):1005-13 doi: 10.1001/jama.2013.167[published Online First: Epub Date]|.

For peer review only

For peer review only - http://bmjopen.bmj.com/site/about/guidelines.xhtml

36. Gupta A, Dadhich JP, Faridi MMA. Breastfeeding and complementary feeding as a public health intervention for child survival in India. Indian J Pediatr 2010;**77**(4):413-18 doi: 10.1007/s12098-010-0049-6[published Online First: Epub Date]|.
37. Census of India 2011. Population Projections for India and States 2001-2026: Report of the Technical Group on Population Projections Constituted by the National Commission on Population. New Delhi: Office of the Registrar General and Census Commissioner, 2006.
38. Renfrew MJ, McCormick FM, Wade A, et al. Support for healthy breastfeeding mothers with healthy term babies. The Cochrane database of systematic reviews 2012;**5**:CD001141-CD41 doi: 10.1002/14651858.CD001141.pub4[published Online First: Epub Date]|.
39. Rollins NC, Bhandari N, Hajeebhoy N, et al. Why invest, and what it will take to improve breastfeeding practices? The Lancet 2016;**387**(10017):491-504 doi: http://dx.doi.org/10.1016/S0140-6736(15)01044-2[published Online First: Epub Date]|.

For peer review only

For peer review only - http://bmjopen.bmj.com/site/about/guidelines.xhtml

Figure 1. Model of the association between village level urbanicity, individual level socio-economic indicators, and breastfeeding practices

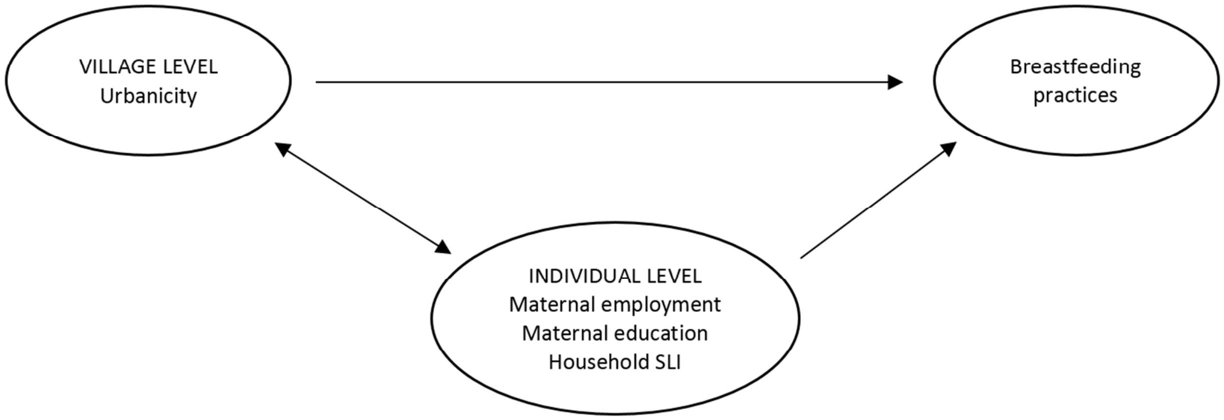

For peer review only

For peer review only - http://bmjopen.bmj.com/site/about/guidelines.xhtml

Figure 2. Flowchart of how samples for the two breastfeeding indicators were reached

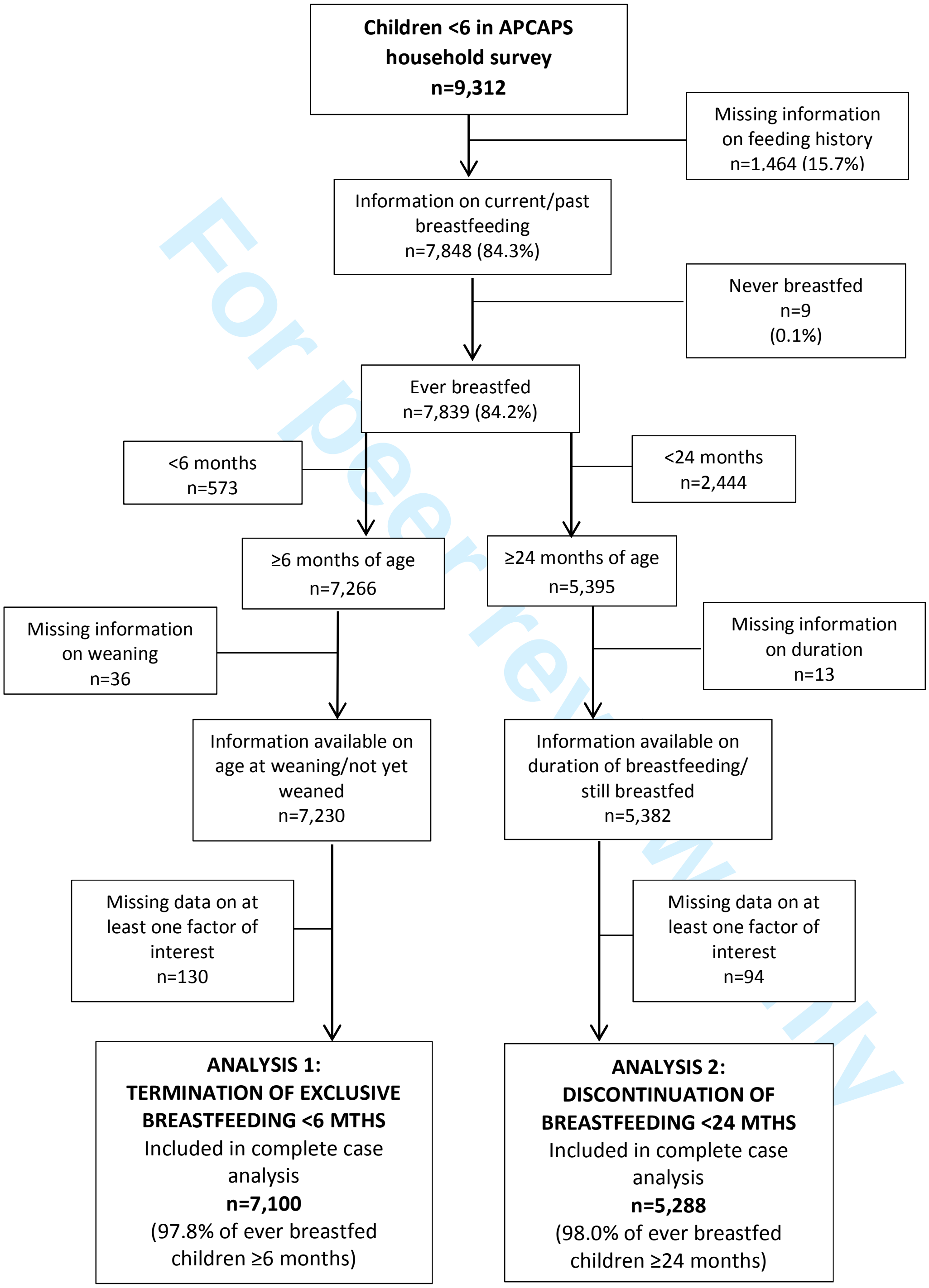

For peer review only

For peer review only - http://bmjopen.bmj.com/site/about/guidelines.xhtml

Table 1. Characteristics of ever breastfed children under 6 in the APCAPS household survey, by village urbanicity tertile (n=7839)

		**Urbanicity (measured by night-time light intensity)**						**ALL**	
		HIGH tertile 1 (n=4276)		MEDIUM tertile 2 (n=2082)		LOW tertile 3 (n=1476)			
		**n**	**(%)**	**n**	**(%)**	**n**	**(%)**	**n**	**(%)**
**Exclusive breastfeeding ≥6 months[1]**	Yes	2495	(63.5)	1400	(72.8)	915	(66.4)	4810	(66.5)
	No	1436	(36.5)	522	(27.2)	462	(33.6)	2420	(33.5)
**Continued breastfeeding ≥24 months[2]**	Yes	1701	(57.9)	927	(65.0)	717	(70.4)	3345	(62.2)
	No	1236	(42.1)	499	(35.0)	302	(29.6)	2037	(37.8)
**Infant sex**	Male	2189	(51.2)	1069	(51.2)	748	(50.5)	4006	(51.1)
	Female	2087	(48.8)	1013	(48.7)	733	(49.5)	3833	(48.9)
**Age of child at survey**	0-1	1337	(31.3)	650	(31.2)	457	(30.9)	2444	(31.2)
	2-3	1520	(35.5)	718	(34.5)	535	(36.1)	2773	(35.4)
	4-5	1419	(33.2)	714	(34.3)	489	(33.0)	2622	(33.4)
**Birth order**	1	1824	(43.1)	883	(42.7)	607	(41.0)	3314	(42.6)
	2	1667	(39.4)	798	(38.6)	588	(39.8)	3053	(39.2)
	≥3	743	(17.5)	389	(18.8)	284	(19.2)	1416	(18.2)
	missing	*42*		*12*		*2*		56	
	mean (SD)	1.79	(0.85)	1.80	(0.85)	1.82	(0.85)	1.80	(0.85)
**Age of mother at birth**	<20	737	(17.3)	364	(17.6)	276	(18.6)	1377	(17.6)
	20-24	2410	(56.5)	1238	(59.6)	862	(57.7)	4510	(57.7)
	25-29	917	(22.1)	393	(19.0)	288	(20.2)	1598	(20.2)
	30+	198	(44.6)	78	(3.8)	54	(3.6)	330	(4.2)
	missing	*14*		*9*		*1*		*24*	
	mean (SD)	22.9	(3.6)	22.7	(3.4)	22.7	(3.5)	22.8	(3.5)
**Family structure**	Nuclear	2866	(67.6)	1306	(63.7)	942	(64.0)	5114	(65.9)
	Joint/extended	1371	(32.4)	745	(36.3)	530	(36.0)	2646	(34.1)
	missing	*39*		*31*		*9*		79	
**Maternal education**	No formal schooling	1054	(24.7)	663	(32.0)	558	(37.7)	2275	(29.1)
	Primary	880	(20.6)	390	(18.8)	284	(19.2)	1554	(19.9)
	Secondary+	2329	(54.6)	1022	(49.3)	638	(43.1)	3989	(51.0)
	missing	*13*		*7*		*1*		*21*	
**Maternal employment**	Not working	3144	(73.8)	*1342*	(64.7)	*815*	(55.0)	5301	(67.8)
	Working	1119	(26.2)	*733*	(35.3)	*666*	(45.0)	2518	(32.2)
	missing	*13*		*7*		*0*		*20*	
**Standard of living (SLI) index**	Poorest	611	(14.3)	318	(15.3)	242	(16.3)	1171	(14.9)
	Poorer	736	(17.2)	422	(20.3)	288	(19.4)	1446	(18.5)
	Middle	816	(19.1)	458	(22.0)	378	(25.5)	1652	(21.1)
	Richer	964	(22.5)	497	(23.9)	314	(21.2)	1775	(22.6)
	Richest	1148	(26.9)	386	(18.5)	259	(17.5)	1793	(22.9)
	missing	*1*		*1*		*0*		*2*	

[1]Restricted to ever breastfed infants aged at least 6 months not yet weaned/with age at weaning (n=7230)

[2]Restricted to ever breastfed infants aged at least 24 months still breastfed/with age at cessation of breastfeeding (n=5382)

[3]chi-square test of independence for association with urbanicity

For peer review only

For peer review only - http://bmjopen.bmj.com/site/about/guidelines.xhtml

Table 2. Results of multilevel logistic regression models for the association between urbanicity or individual/household socio-economic factors and termination of exclusive breastfeeding <6 months, among ever-breastfed children under 6 in the APCAPS household survey[1]

				Termination of exclusive breastfeeding <6 months (n=7100)											
				Unadjusted		Model 1 (null)		Model 2 (L1 confounders + L2 SES)		Model 3 (L1 confounders + L3 urbanicity)		Model 4 (L1 confounders + L2 SES + L3 urbanicity)	
		n	(%)	OR	(95% CI)	OR	(95% CI)	OR	(95% CI)	OR	(95% CI)	OR	(95% CI)
FIXED EFFECTS													
**Maternal education**	No formal schooling	615	(29.1)	**ref**				ref				ref	
	Primary	534	(38.6)	3.42	(2.23, 5.25)			3.37	(2.13, 5.31)			3.36	(2.13, 5.30)
	Secondary+	1231	(38.2)	1.82	(1.29, 2.57)			1.69	(1.12, 2.54)			1.69	(1.12, 2.54)
**Maternal employment**	Not working	1584	(33.5)	ref				ref				ref	
	Working	796	(33.7)	0.94	(0.69, 1.28)			1.43	(1.00, 2.03)			1.43	(1.00-2.04)
**Standard of living (SLI) index**	Poorest	280	(26.8)	ref				ref				ref	
	Poorer	432	(32.9)	1.80	(1.07, 3.02)			1.65	(0.97, 2.81)			1.66	(0.98, 2.82)
	Middle	525	(35.1)	2.24	(1.36, 3.71)			2.08	(1.24, 3.50)			2.09	(1.24, 3.51)
	Richer	564	(35.1)	2.19	(1.33, 3.59)			1.98	(1.17, 3.34)			1.99	(1.18, 3.35)
	Richest	579	(35.3)	2.22	(1.35, 3.65)			2.11	(1.22, 3.63)			2.11	(1.23, 3.64)
	trend[2] (p value)			*0.004*				*0.003*				*0.015*	
Urbanicity	Low	459	(33.7)	ref								ref	
	Medium	517	(27.5)	0.49	(0.15, 1.56)					0.48	(0.15, 1.54)	0.48	(0.15, 1.51)
	High	1404	(36.4)	1.10	(0.35, 3.45)					1.09	(0.34, 3.43)	1.04	(0.34, 3.20)
	trend[2] (p value)			*0.87*						*0.89*		*0.91*	
RANDOM EFFECTS													
Level 2 (mothers)	*variance (SE)*					13.6194	(1.2202)	13.9036	(1.2454)	13.9888	(1.2531)	13.9072	1.2456
	PCV (compared to null)[3] (%)					ref		-2.09%		-2.71%		-2.11%	
Level 3 (villages)	*variance (SE)*					1.4949	(0.4712)	1.4429	(0.4574)	1.3870	(0.4427)	1.3200	0.4235
	PCV (compared to null)[3] (%)					ref		3.48%		7.22%		11.70%	
	PCV (compared to model 2)[4]					-		ref		-		8.52%	

[1]All ORs calculated using multilevel modelling and complete case sample (see Figure 2)
[2]Test for trend: p value including variable as linear
[3]Proportional Change in Variance ((model 1 variance – model X variance)/model 1 variance)*100%
[4]Proportional Change in Variance ((model 2 variance – model 4 variance)/model 2 variance)*100%

For peer review only

For peer review only - http://bmjopen.bmj.com/site/about/guidelines.xhtml

Table 3. Results of multilevel logistic regression models for the association between urbanicity or individual/household socio-economic factors and discontinuation of continued breastfeeding<24 months, among ever-breastfed children under 6 in the APCAPS household survey[1]

		Discontinuation of breastfeeding <24 months (n=5288)													
				**Unadjusted**		**Model 1 (null)**		**Model 2 (L1 confounders + L2 SES)**		**Model 3 (L1 confounders + L3 urbanicity)**		**Model 4 (L1 confounders + L2 SES + L3 urbanicity)**	
		**n**	**(%)**	**OR**	**(95% CI)**	**OR**	**(95% CI)**	**OR**	**(95% CI)**	**OR**	**(95% CI)**	**OR**	**(95% CI)**
FIXED EFFECTS													
**Maternal education**	No formal schooling	521	(30.7)	ref				ref				ref	
	Primary	395	(37.3)	1.48	(1.13, 1.94)			1.21	(0.88, 1.65)			1.21	(0.88, 1.65)
	Secondary+	1079	(42.5)	2.28	(1.82, 2.87)			1.63	(1.23, 2.16)			1.62	(1.22, 2.15)
**Maternal employment**	Not working	1384	(41.3)	ref				ref				ref	
	Working	611	(31.6)	0.55	(0.45, 0.67)			0.77	(0.60, 0.99)			0.78	(0.61, 0.99)
**Standard of (SLI) index**	Poorest	271	(34.6)	ref				ref				ref	
	Poorer	370	(37.3)	1.38	(0.99, 1.91)			1.18	(0.82, 1.69)			1.17	(0.82, 1.68)
	Middle	374	(33.1)	1.07	(0.78, 1.48)			0.86	(0.60, 1.24)			0.86	(0.60, 1.23)
	Richer	467	(39.4)	1.5	(1.09, 2.06)			1.05	(0.73, 1.51)			1.04	(0.73, 1.50)
	Richest	513	(42.9)	1.75	(1.28, 2.41)			1.09	(0.74, 1.59)			1.08	(0.74, 1.57)
	trend[2] (p value)			*0.001*				*0.027*				*0.915*	
Urbanicity	Low	290	(29.5)	ref						ref		ref	
	Medium	494	(34.6)	1.40	(0.72, 2.71)					1.45	(0.71, 2.96)	1.35	(0.66, 2.79)
	High	1213	(42.2)	2.74	(1.42, 5.28)					2.96	(1.45, 6.05)	2.64	(1.29, 5.42)
	trend[2] (p value)			*0.003*						*0.003*		*0.008*	
RANDOM EFFECTS													
Level 2 (mothers)	*variance (SE)*					3.2070	(0.4847)	4.1838	(0.6347)	4.2895	(0.6445)	4.1793	(0.6342)
	PCV (compared to null)[3] (%)					ref		-30.46%		-33.75%		-30.32%	
Level 3 (villages)	*variance (SE)*					0.6036	(0.1966)	0.6677	(0.2221)	0.4942	(0.1718)	0.5029	(0.1739)
	PCV (compared to null)[3] (%)					ref		-10.62%		18.12%		16.68%	
	PCV (compared to model 2)[4]					-		ref		-		24.68%	

[1]All ORs calculated using multilevel modelling and complete case sample (see Figure 2)

[2]Test for trend: p value including variable as linear

[3]Proportional Change in Variance ((model 1 variance – model X variance)/model 1 variance)*100%

[4]Proportional Change in Variance ((model 2 variance – model 4 variance)/model 2 variance)*100%

For peer review only

For peer review only - http://bmjopen.bmj.com/site/about/guidelines.xhtml

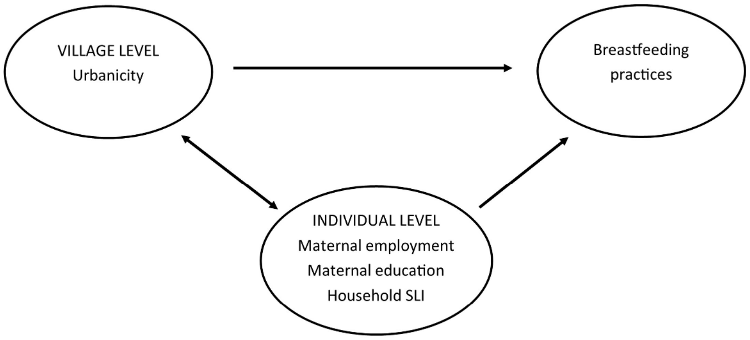

Figure 1. Model of the association between village level urbanicity, individual level socio-economic indicators, and breastfeeding practices

209x297mm (300 x 300 DPI)

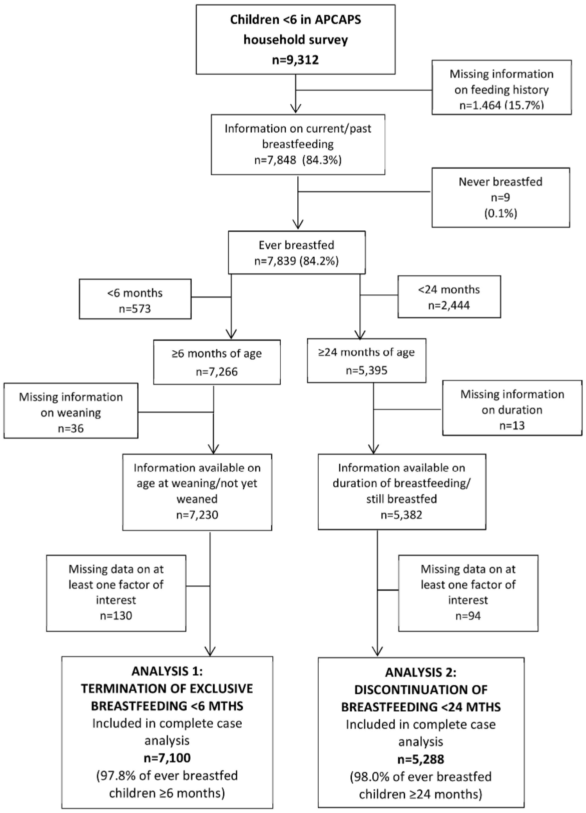

Figure 2. Flowchart of how samples for the two breastfeeding indicators were reached

297x420mm (300 x 300 DPI)

Supplementary table 1. Characteristics of children under 6 in the APCAPS household survey, by missing/non-missing information on breastfeeding history (n=9,312)

		Children <6 years (n=9,312) Information on current/past breastfeeding available				
		Yes (non-missing) (n=7,848)		No (missing) (n=1,464)		
		**n**	**(%)**	**n**	**(%)**	*p value*
INDIVIDUAL LEVEL						
**Infant sex**	Male	4011	(51.1)	773	(52.8)	*0.23*
	Female	3837	(48.9)	691	(47.2)	
**Age of infant**	0-1	2445	(31.2)	445	(30.4)	
	2-3	2776	(35.4)	501	(34.2)	*0.36*
	4-5	2627	(33.5)	518	(35.4)	
HOUSEHOLD LEVEL						
**Number of under 6s in household**	1	3192	(40.7)	616	(42.1)	
	2	3846	(49.0)	702	(48.0)	*0.71*
	≥3	810	(10.3)	146	(10.0)	
**Standard of living (SLI) index**	Poorest	1172	(14.9)	145	(15.5)	
	Poorer	1447	(18.4)	176	(18.8)	
	Middle	1654	(21.1)	211	(22.5)	*0.37*
	Richer	1777	(22.6)	185	(19.8)	
	Richest	1796	(22.9)	219	(23.4)	
	Missing	*2*		*528*		
VILLAGE LEVEL						
**Night-time light intensity (NTLI)**	Low	1484	(18.9)	271	(18.5)	
	Medium	2086	(26.6)	353	(24.1)	*0.09*
	High	4278	(54.5)	840	(57.4)	

For peer review only

Supplementary figure 1. Questions on breastfeeding practices from APCAPS cross-sectional household survey

12. Health Profile of the child (≤5 years)								
(12.1) **Name of the Child**	(12.2) **Colostrum Intake** (1. Yes; 2. No)	(12.3) **Total Duration of Breast Feeding** (in months)	(12.4) **Age of child at onset of weaning** (in months)	(12.5) **Immunization** (1. Completed; 2. Partial; 3. None)	(12.6) **Normal Healthy Child** (1. Yes; 2. No)	**Child Supplemented during** (1. Yes; 2. No; 3. N.A.)		
						(12.7.1) **Infancy**	(12.7.2) **Childhood**	(12.7.3) **Current**

STROBE Statement—checklist of items that should be included in reports of observational studies

	Item No	Recommendation	Location in manuscript
**Title and abstract**	1	(*a*) Indicate the study's design with a commonly used term in the title or the abstract	*In abstract (cross-sectional survey), page 2*
		(*b*) Provide in the abstract an informative and balanced summary of what was done and what was found	*Done, page 2.*
**Introduction**			
Background/rationale	2	Explain the scientific background and rationale for the investigation being reported	*Done, pages 4-5.*
Objectives	3	State specific objectives, including any prespecified hypotheses	*Done (objective in Introduction page 5, hypothesis in Methods page 8)*
**Methods**			
Study design	4	Present key elements of study design early in the paper	*Done, pages 5-6.*
Setting	5	Describe the setting, locations, and relevant dates, including periods of recruitment, exposure, follow-up, and data collection	*Done, pages 5-6.*
Participants	6	(*a*) *Cohort study*—Give the eligibility criteria, and the sources and methods of selection of participants. Describe methods of follow-up<br>*Case-control study*—Give the eligibility criteria, and the sources and methods of case ascertainment and control selection. Give the rationale for the choice of cases and controls<br>*Cross-sectional study*—Give the eligibility criteria, and the sources and methods of selection of participants	*Done, pages 5-6, 8-9 (please also see Fig 2)*
		(*b*) *Cohort study*—For matched studies, give matching criteria and number of exposed and unexposed<br>*Case-control study*—For matched studies, give matching criteria and the number of controls per case	
Variables	7	Clearly define all outcomes, exposures, predictors, potential confounders, and effect modifiers. Give diagnostic criteria, if applicable	*Done, pages 6-9.*
Data sources/ measurement	8*	For each variable of interest, give sources of data and details of methods of assessment (measurement). Describe comparability of assessment methods if there is more than one group	*Done, pages 6-8.*
Bias	9	Describe any efforts to address potential sources of bias	*Done, pages 7-8, 14-15.*
Study size	10	Explain how the study size was arrived at	*N/A (secondary analysis)*
Quantitative variables	11	Explain how quantitative variables were handled in the analyses. If applicable, describe which groupings were chosen and why	*Done, pages 6-9.*
Statistical methods	12	(*a*) Describe all statistical methods, including those used to control for confounding	*Done, pages 8-9.*
		(*b*) Describe any methods used to examine subgroups and interactions	*Done, page 9.*
		(*c*) Explain how missing data were addressed	*Done (end of para 1 'Statistical analysis' page 8, also see Supplementary Table 1)*
		(*d*) *Cohort study*—If applicable, explain how loss to follow-up was	

For peer review only - http://bmjopen.bmj.com/site/about/guidelines.xhtml

For peer review only

addressed<br>*Case-control study*—If applicable, explain how matching of cases and controls was addressed<br>*Cross-sectional study*—If applicable, describe analytical methods taking account of sampling strategy	*N/A*
(*e*) Describe any sensitivity analyses	

Continued on next page

For peer review only

For peer review only - http://bmjopen.bmj.com/site/about/guidelines.xhtml

**Results**			**Location in manuscript**
Participants	13*	(a) Report numbers of individuals at each stage of study—eg numbers potentially eligible, examined for eligibility, confirmed eligible, included in the study, completing follow-up, and analysed	*Done, page 9 and Figure 2.*
		(b) Give reasons for non-participation at each stage	*Done, Figure 2.*
		(c) Consider use of a flow diagram	*Provided (Figure 2).*
Descriptive data	14*	(a) Give characteristics of study participants (eg demographic, clinical, social) and information on exposures and potential confounders	*Done, Table 1.*
		(b) Indicate number of participants with missing data for each variable of interest	*Done, Table 1.*
		(c) *Cohort study*—Summarise follow-up time (eg, average and total amount)	
Outcome data	15*	*Cohort study*—Report numbers of outcome events or summary measures over time	
		Case-control study—Report numbers in each exposure category, or summary measures of exposure	
		Cross-sectional study—Report numbers of outcome events or summary measures	*Done, Table 1.*
Main results	16	(*a*) Give unadjusted estimates and, if applicable, confounder-adjusted estimates and their precision (eg, 95% confidence interval). Make clear which confounders were adjusted for and why they were included	*Done: pages 9-12, Tables 2 and 3.*
		(*b*) Report category boundaries when continuous variables were categorized	*Tables 2 and 3.*
		(*c*) If relevant, consider translating estimates of relative risk into absolute risk for a meaningful time period	
Other analyses	17	Report other analyses done—eg analyses of subgroups and interactions, and sensitivity analyses	*Done, pages 10-12.*
**Discussion**			
Key results	18	Summarise key results with reference to study objectives	*Done, pages 12-13.*
Limitations	19	Discuss limitations of the study, taking into account sources of potential bias or imprecision. Discuss both direction and magnitude of any potential bias	*Done, pages 14-15.*
Interpretation	20	Give a cautious overall interpretation of results considering objectives, limitations, multiplicity of analyses, results from similar studies, and other relevant evidence	*Done, pages 15-16.*
Generalisability	21	Discuss the generalisability (external validity) of the study results	*Done, pages 15-16.*
**Other information**			
Funding	22	Give the source of funding and the role of the funders for the present study and, if applicable, for the original study on which the present article is based	*Done, page 17.*

*Give information separately for cases and controls in case-control studies and, if applicable, for exposed and unexposed groups in cohort and cross-sectional studies.

Note: An Explanation and Elaboration article discusses each checklist item and gives methodological background and published examples of transparent reporting. The STROBE checklist is best used in conjunction with this article (freely available on the Web sites of PLoS Medicine at http://www.plosmedicine.org/, Annals of Internal Medicine at http://www.annals.org/, and Epidemiology at http://www.epidem.com/). Information on the STROBE Initiative is available at www.strobe-statement.org.

For peer review only

For peer review only - http://bmjopen.bmj.com/site/about/guidelines.xhtml
